# The role of RIPK1 mediated cell death in acute on chronic liver failure

**DOI:** 10.1038/s41419-021-04442-9

**Published:** 2021-12-17

**Authors:** Takayuki Kondo, Stewart Macdonald, Cornelius Engelmann, Abeba Habtesion, Jane Macnaughtan, Gautam Mehta, Rajeshwar P. Mookerjee, Nathan Davies, Marco Pavesi, Richard Moreau, Paolo Angeli, Vicente Arroyo, Fausto Andreola, Rajiv Jalan

**Affiliations:** 1 Liver Failure Group, Institute for Liver and Digestive Health, University College London, London, UK; 2 Department of Gastroenterology, Graduate School of Medicine, Chiba University, Chiba, Japan; 3 Section Hepatology, Clinic for Gastroenterology and Rheumatology, University Hospital Leipzig, Leipzig, Germany; 4 Department of Hepatology and Gastroenterology, Campus Virchow-Klinikum and Charité Campus Mitte, Charité - Universitaetsmedizin Berlin, Berlin, Germany; 5 The Roger Williams Institute of Hepatology, Foundation for Liver Research, London, UK; 6 European Foundation of the study of Chronic Liver Failure (EF-CLIF), Barcelona, Spain; 7 Inserm, U1149, Centre de Recherche sur l’Inflammation (CRI), Clichy, Paris, France; 8 UMRS1149, Université de Paris, Paris, France; 9 Assistance Publique-Hôpitaux de Paris, Service d’Hépatologie, Hôpital Beaujon, Clichy, France; 10 Unit of Internal Medicine and Hepatology (UIMH), Department of Medicine - DIMED University of Padova, Padova, Italy

**Keywords:** Hepatitis, Liver diseases

## Abstract

Acute-on-chronic liver failure (ACLF) is characterized predominantly by non-apoptotic forms of hepatocyte cell death. Necroptosis is a form of programmed lytic cell death in which receptor interacting protein kinase (RIPK) 1, RIPK3 and phosphorylated mixed lineage kinase domain-like (pMLKL) are key components. This study was performed to determine the role of RIPK1 mediated cell death in ACLF. RIPK3 plasma levels and hepatic expression of RIPK1, RIPK3, and pMLKL were measured in healthy volunteers, stable patients with cirrhosis, and in hospitalized cirrhotic patients with acutely decompensated cirrhosis, with and without ACLF (AD). The role of necroptosis in ACLF was studied in two animal models of ACLF using inhibitors of RIPK1, necrostatin-1 (NEC-1) and SML2100 (RIPA56). Plasma RIPK3 levels predicted the risk of 28- and 90-day mortality (AUROC, 0.653 (95%CI 0.530–0.776), 0.696 (95%CI 0.593–0.799)] and also the progression of patients from no ACLF to ACLF [0.744 (95%CI 0.593–0.895)] and the results were validated in a 2^nd^ patient cohort. This pattern was replicated in a rodent model of ACLF that was induced by administration of lipopolysaccharide (LPS) to bile-duct ligated rats and carbon tetrachloride-induced fibrosis mice administered galactosamine (CCL_4_/GalN). Suppression of caspase-8 activity in ACLF rodent model was observed suggesting a switch from caspase-dependent cell death to necroptosis. NEC-1 treatment prior to administration of LPS significantly reduced the severity of ACLF manifested by reduced liver, kidney, and brain injury mirrored by reduced hepatic and renal cell death. Similar hepato-protective effects were observed with RIPA56 in a murine model of ACLF induced by CCL_4_/GalN. These data demonstrate for the first time the importance of RIPK1 mediated cell death in human and rodent ACLF. Inhibition of RIPK1 is a potential novel therapeutic approach to prevent progression of susceptible patients from no ACLF to ACLF.

## Introduction

The most common hospital presentation of patients with cirrhosis is an acute decompensation (AD) with jaundice, gastrointestinal bleeding, bacterial infection, acute onset of ascites or hepatic encephalopathy (HE), alone or in combination [1, 2]. Acute on chronic liver failure (ACLF) occurs in about 30% of these patients and is defined by the occurrence of hepatic and extra-hepatic organ failures associated with a 28-day mortality of about 30%. There are currently no clinically available therapies [2–7]. The PIRO concept (Predisposition, Injury, Response and Organ Failures) has been proposed to categorize ACLF patients into distinct pathophysiological and prognostic groups [8]. We have previously shown that AD is associated with marked elevation in circulating markers of hepatic cell death and this increases further as ACLF develops. Importantly, the data showed that the mode of cell death evolves from predominantly apoptosis to other non-apoptotic forms of cell death in ACLF, which are known to be more immunogenic [9–12].

Necroptosis is a form of programmed necrosis that shares the same upstream receptors as apoptosis [tumor necrosis factor receptor 1 (TNFR1), TNFR2, TNF-related apoptosis including ligand (TRAIL) 1-2 and Fas]. However, under low ATP conditions or caspase inhibition, stimulation of these death receptors leads not to apoptosis but to cellular swelling and leakage of cellular contents, which morphologically resembles necrosis. The pathway leading to necroptosis is an area of intense research but it is clear that receptor-interacting protein kinase 1 (RIPK1) and RIPK3 are central to the process [13]. Upon death receptor stimulation, activation of caspase-8 results in RIPK1 and RIPK3 cleavage, leading to apoptosis; however, inhibition of caspase-8 preserves the integrity of RIPK1 and RIPK3 allowing the formation of a multimolecular complex, known as the necrosome. Classically, the necrosome complex formation depends on the interaction between RIPK1 and RIPK3 through the RHIM domain upon phosphorylation at their kinase domains.

RIPK3 phosphorylation is known to be dependent on both the kinase activity of RIPK1 and its own kinase activity [14]. The final step of necrosome formation is RIPK3-mediated phosphorylation of the mixed lineage kinase like protein (MLKL); once phosphorylated, MLKL undergoes oligomerisation and translocation to the cell membrane, triggering not only the NLRP3 inflammasome [15] but also resulting in cell swelling, rupture and release of damage-associated molecular patterns (DAMPs) such as fragmented chromatin (mono- and poly-nucleosomes, core histones). These can drive further inflammasome activation leading to death of the neighboring cells and can potentially modulate extra hepatic organ failure [16–21].

A number of studies have addressed the role of necroptosis in various models of acute and chronic liver diseases [22]. Recent studies suggest a multifaceted role of RIPK3 in multiple causes of liver injury [23–25] whereas in the context of high-fat diet induced liver injury, RIPK3 seems to exert a protective role [16]. The role of RIPK3-dependent necroptosis is not known in the pathogenesis of ACLF.

The aims of this study were to assess whether RIPK1 mediated cell death is a feature of ACLF and to define its potential role by pharmacological blockade of RIPK1 impacting on the necroptotic pathway using necrostatin-1 (NEC-1) or RIPA56 in rodent models of ACLF. Therefore, we first evaluated the RIPK3 levels, a marker of necroptosis in patients with ACLF and then in a rodent model. The role of RIPK1 mediated cell death in ACLF was then determined using NEC-1 or RIPA56 in two different animal models.

## Material and methods

### Study design

Patient samples for the derivation cohort were obtained from the CANONIC study and for the validation cohort from prospectively collected bio-banked material at Royal Free Hospital as part of the DASIMAR study. The study was carried out in accordance with the principles of good clinical practice and Declaration of Helsinki (1951). Informed written consent was obtained from all subjects.

All animal experiments were conducted according to the Home Office guidelines under the UK Animals in Scientific Procedures Act 1986 with approval of the ethical committee for animal care of University College London. Rodents were used to generate both an in vitro necroptosis model and an in vivo model of ACLF to improve understanding of the role of necroptosis in ACLF and define the role of necroptosis using RIPK1 inhibitors, NEC1 and its novel derivative RIPA56, as a treatment(s). RIPA56 was chosen as being more selective and potent (60-fold) in inhibiting RIPK1 kinase activity and unable to inhibit IDO activity, hence avoiding the off-target effects shown by NEC1 [26].

### Human studies

#### CANONIC study (derivation cohort)—cohort 1

Samples of patients with AD and ACLF were obtained from subjects recruited within the CANONIC study [6]. All patients were enrolled prospectively, and the study was designed specifically to define the clinical and prognostic features of ACLF. In total 1343 patients who were hospitalized with an acute decompensation of cirrhosis (bacterial infection, large volume ascites, gastrointestinal hemorrhage, hepatic encephalopathy, alone or in combination) were enrolled in 29 Hepatology centers across 8 countries [6]. Of the initial cohort, 311 patients with plasma samples were randomly selected for analysis (Fig. S1). Baseline characteristics of patients included in this study, which are displayed in Table 1 and Table S1, closely reflect the patients described in the CANONIC study [6]. RIPK3 levels measured in the current study were correlated with markers for cell death [K18 and cK18 - MacDonald S et al. [9]] and inflammation [sCD163, IL6, IL8, IL1ra – Claria J et al. [5]] which were analyzed in previous studies on the same cohort.

**Table 1:** Baseline characteristics stratified by the presence or absence of ACLF at enrollment (derivation cohort).

	No ACLF (*n* = 187)	ACLF (*n* = 124)	*p* values
Age (years)	58 (50–67)	56 (49–64)	0.167
Male (*n*, %)	129 (69.4)	85 (68)	0.804
Etiology (*n*, %)			
Alcohol	**86 (45.9)**	**70 (56.5)**	**0.011**
Alcohol + HCV	64 (34.2)	55 (44.4)	0.339
HCV	50 (26.7)	25 (20.2)	0.316
Non-alcoholic steatohepatitis	11 (5.9)	6 (4.8)	0.803
Other	14 (7.5)	7 (5.6)	0.647
Active alcoholism at enrollment (*n*, %)	**17 (9.1)**	**29 (23.4)**	**0.003**
Ascites (*n*, %)	61 (32.6)	83 (66.9)	0.066
Gastrointestinal bleeding (*n*, %)	22 (11.8)	14 (14.3)	>0.999
Bacterial infection (*n*, %)	19 (32.2)	15 (31.9)	>0.999
Organ failure (*n*, %)			
Liver	**14 (7.5)**	**47 (37.9)**	**<0.001**
Kidney	-	77 (62.1)	-
Brain	**7 (3.7)**	**26 (20.9)**	**<0.001**
Coagulation	**4 (2.1)**	**27 (21.8)**	**<0.001**
Cardiac	**1 (0.5)**	**19 (15.3)**	**<0.001**
Respiratory	**1 (0.5)**	**10 (8)**	**0.001**
Laboratory values			
White blood cell (× 10^9^/L)	**5.6 (4.1–8.9)**	**9 (5.8–13.8)**	**<0.001**
Bilirubin (mg/dL)	**2.7 (1.5–4.9)**	**6 (1.9–16.8)**	**<0.001**
Prothrombin time (international normalized ratio)	**1.5 (1.3–1.8)**	**1.7 (1.4–2.3)**	**<0.001**
Albumin (g/dL)	2.8 ± 0.7	2.9 ± 0.7	0.608
Creatinine (mg/dL)	**1 (0.8-1.3)**	**2.2 (0.9–3.2)**	**<0.001**
Sodium (mmol/L)	**137 (132–140)**	**134 (130–139)**	**0.004**
Platelets (10^9^/L)	86.6 (56.5–127.5)	77 (51–126)	0.359
Scores			
MELD	**16 (13–21)**	**26 (21–32.5)**	**<0.001**
MELD Na	**18 (14–23)**	**29 (23–34)**	**<0.001**
Child-Pugh score	**9 (8–11)**	**11 (9–12.5)**	**<0.001**
CLIF-OFs	**7 (6–8)**	**10 (9–11)**	**<0.001**
Outcome			
28-day mortality (*n*, %)	**9 (5.9)**	**22 (22.4)**	**<0.001**
90-day mortality (*n*, %)	**22 (14.4)**	**35 (35.7)**	**<0.001**

Continues data are expressed as mean ± standard deviation or median (Q1–Q3), as appropriate. Categorical data are expressed as *n* (%)

*ACLF* acute on chronic liver failure, *CLIF-OFs* chronic liver failure-organ failure score, *HCV* hepatitis C virus, *MELD* model for end-stage liver disease.

#### DASIMAR study (validation cohort)—cohort 2

Bio-banked plasma samples and associated clinical data were obtained for 106 patients with decompensated cirrhosis from the multi-center, prospective, observational DASIMAR study (NCT01071746) (Fig. S1), designed to identify biomarkers of ACLF. Inclusions: Cirrhotic patients admitted to the hospital with acute deterioration associated with a precipitating illness. Exclusions: Patients were excluded if they were admitted for reasons other than acute decompensation of cirrhosis, had evidence of extra-hepatic malignancy or hepatocellular carcinoma), patients who had undergone major surgery or have unsolved surgical problems and pregnancy. Baseline characteristics are displayed in Tables S2 and S3.

#### Cohort 3

Samples from healthy volunteers and those with stable cirrhosis (UCL Biobank Ethical Review Committee approval number NC.2017.16) were obtained from archived bio-banked material at Royal Free Hospital (London, UK).

#### Cohort 4

Liver sections from patients with alcoholic hepatitis with or without ACLF were obtained from the histology department of the Royal Free Hospital in London (UCL Biobank Ethical Review Committee approval number NC.2017.10) (baseline characteristics Table S4).

Definitions for acute decompensation and ACLF are provided in the supplementary information.

### Measurement of human plasma RIPK3

Plasma samples from cohort 1 and 2 were obtained by centrifugation of human blood at 2000 rpm for 10 min, and storage at −80 °C within 4 h of collection. RIPK3 levels were measured in duplicate in 1:40 diluted plasma samples using a commercially available enzyme-linked immunosorbent assay (ELISA) as per manufacturer’s protocol (Human RIPK3 ELISA, CUSABIO, Wuhan, China). After addition of chromogenic substrate, absorbance was measured at 450 nm using a plate reader (FLUOstar Omega, BMG Labtech, UK).

### Animal studies

Two animal models were used for this study. The sample size was estimated based on previous experiments [10, 11]. Investigators for analyses were not blinded.

### Bile duct ligation plus Lipopolysaccharide (LPS) rat model of ACLF

#### Animals

Male Sprague-Dawley rats (Charles River UK, Margate, UK) (~300 g b.w., 8–10 weeks-old) underwent either sham operation or bile duct ligation (BDL) to induce advanced fibrosis as described previously [27]; ACLF was induced by intraperitoneal injection of *Klebsiella pneumoniae* LPS (Sigma, UK) (0.03 mg/ml in saline) 28 days after BDL.

#### Necrostatin 1

Nec1 (Calbiochem, Sigma Aldrich, UK) was prepared as follows. Briefly, Nec-1 was redissolved in N-2 methyl-pyrrolidone (NMP) to make a 200 mg/ml stock solution further diluted to an intermediate concentration of 40 mg/ml in NMP. The 40 mg/ml solution was finally diluted to a working concentration of 1 mg/ml in a solution of 30% (2-hydroxypropyl) β-cyclodextrin (Sigma, Cat No H107)/0.5% Citric acid. Vehicle controls consisted of NMP and 30%(2-hydroxypropyl) β-cyclodextrin/0.5% Citric acid (1:40 ratio).

#### Study design

Animals underwent BDL surgery and were given 4 weeks to develop advanced fibrosis and portal hypertension [27]. The number per treatment group was determined based on liver function test with two-tailed tests with type I and II errors set at 0.05 and 0.20, respectively.

#### Bile-duct ligation group

The animals were assigned randomly into 3 groups 4-weeks after bile-duct ligation.

- **BDL** + **Vehicle (*n*** = **4):** BDL rats received an injection of intraperitoneal (i.p.) vehicle control and were euthanised 6 h later under general anesthesia.
- **BDL** + **Vehicle** + **LPS (*n*** = **9):** BDL rats received an i.p. injection of vehicle control followed an i.p. injection of LPS (0.025 mg/kg), 3 h later. Animals were euthanised 3 h after LPS injection (or whenever comatose).
- **BDL** + **Nec-1** + **LPS (*n*** = **8):** This group of BDL rats received an i.p. injection of 3.3 mg/kg Nec-1 followed, by an i.p injection of LPS (0.025 mg/kg), 3 h later. Animals were euthanised 3 h after LPS.

#### Control group

Sham operated rats (*n* = 4) were also included in the analyses.

### Carbon tetrachloride (CCl_4_) plus Galactosamine (GalN) mouse model of ACLF

#### Animals

For this model C57BL/6 male mice (~30 g b.w; 8–10 weeks-old) were randomly assigned to four groups,

- **Vehicle (*n*** = **8):** gavaged with olive oil twice weekly for 6 weeks.
- **CCl**_**4**_ **(*n*** = **8):** gavaged with carbon tetrachloride (CCl_4_ 0.5 mg/ml in olive oil—dose 0.5 ml/kg) twice weekly for 6 weeks, to induce a chronic liver injury as described previously.
- **CCl**_**4**_ + **GalN (*n*** = **8):** Three days after the last dose of CCl_4_ mice were intraperitoneally injected with GalN 1000 mg/kg (Sigma, UK) in saline. All animals were sacrificed 48 h after GalN injection under general anesthesia.
- **CCl**_**4**_ + **GalN** + **RIPA56 (*n*** = **8):** RIPA56 (SML2100, Sigma UK) was prepared as described for Nec-1. RIPA 56 (3 mg/kg, i.p) was injected twice daily with the first injection 1 h after GalN injection. All animals survived the 48 h observational period.

### Animal blood and tissue handling, brain water determination

Plasma was separated from arterial blood collected in heparin or EDTA tubes by refrigerated centrifugation (3500 rpm, 10 min) and stored at −80 °C until assessed for biochemistry, and other endopoints. Liver, brain, and kidney tissues were snap frozen and formalin-fixed for further analysis and histological assessment, respectively.

Immediately after termination, the whole brain was removed, and frontal cortex specimens (50 mm^2^) collected. The brain water content was calculated using the formula: Brain water (%) = (1−dry weight / wet weight) × 100.

### Biochemistry and determination of plasma markers

Clinical biochemistry was assessed using the COBAS system (COBAS Integra 400 plus, Roche Diagnostics).

Plasma RIPK3 and Histone 3 levels were quantified using ELISAs [Rat RIPK3 ELISA kit (Wuhan Fine Biotech, China), EpiQuick total histone H3 quantification kit (Epigenetek, USA) respectively], according to manufacturer protocols. Absorbance was determined using a plate-reader (FLUOstar Omega, BMG Labtech, UK).

### Caspase assays

Caspase 3/7 and 8 activities were measured in liver extracts using Caspase-Glo assay kits (Promega) according to a modified protocol [28]. Diluted lysates (10 μg/ml) were mixed with an equal volume of Caspase-Glo reagents (100 μl each) in white 96-well plate and incubated at room temperature for 1 h. Luminescence was measured using a luminometer (FLUOstar Omega, BMG Labtech, UK).

### Immunohistochemistry

Expression of RIPK1, RIPK3, and pMLKL were evaluated by immunohistochemistry in liver sections of patients with alcoholic hepatitis with and without ACLF (Table S4). Histology of rodent model was performed using liver and kidney sections. The paraffin-embedded tissue sections were deparaffinized using xylene and ethanol and endogenous peroxidase activity blocked in 0.3% H_2_O_2_ in water. After antigen retrieval by microwave, slides were blocked in 2.5% normal horse serum and then stained with following primary antibody (complete list of primary antibodies Table S5). Immunoperoxidase staining was completed using RTU Vectastatin universal Elite ABC kit (Vector Laboratories, UK), and 3.3′-diaminobenzidine was used to detect the target proteins. For negative controls primary antibody was replaced with PBS and a rabbit IgG antibody was used as isotype controls (Table S5). Respective images are shown in Fig. S2. RIPK3 antibody validation was performed by using a blocking peptide as shown in Fig. S3.

### In situ detection of cell death by the TUNEL assay

Terminal deoxynucleotidyl transferase biotin-dUTP nick end labeling (TUNEL) staining of deparaffinised, proteinase K-treated liver and kidney sections was performed using the In-Situ Cell Death Detection kit, POD (Roche, UK) as per manufacturer’s protocol.

Specimens were imaged with a Carl Zeiss Axio Scope. A1 microscope equipment with N-Achroplan 10x/0.25 Ph1 and 40x/0.65 Ph2 objectives and Axio Cam Mrc5 (Carl Zeiss Inc., Germany). Quantification of positive areas was assessed by measuring optical density or relative DAB positive areas (%) as appropriate with FIJI Image J software [29, 30].

### Statistical analysis

Human data are expressed as the mean ± standard deviation for normally distributed continuous variables, median and interquartile range (IQR) for not normally distributed continuous variables, and frequencies and percentages for categorical variables. Animal data were expressed as the mean ± standard error of mean. Continuous variables were analyzed using Student’s *t* test, Mann–Whitney U test, or Kruskal–Wallis test, as appropriate. Categorical variables were analyzed by Fisher’s exact test. *p* value of less than 0.05 was considered to be significant.

## Results

### RIPK1 associated cell death is a feature of human ACLF and is related to clinical severity

#### Derivation cohort

**Fig. 1:**
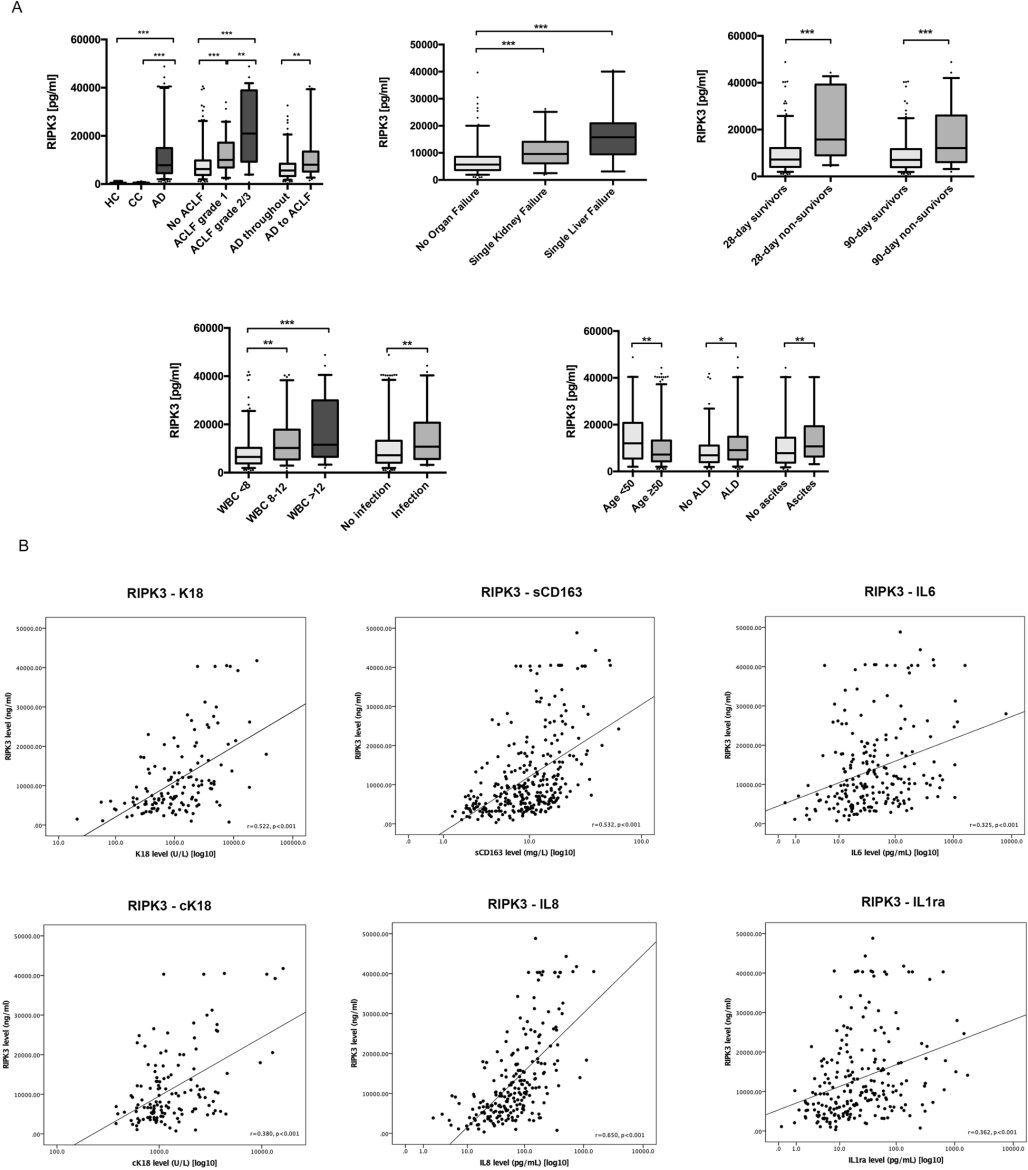
RIPK3 plasma levels derivation cohort. **A** RIPK3 levels in patients with cirrhosis and acute decompensation from the derivation cohort according the severity of liver disease, survival status, inflammation, type of organ failure, and other predisposition factors. Data were analyzed using Student’s *t* test, Mann–Whitney U test or Kruskal–Wallis test followed by Fisher’s Least Significant Difference test as appropriate. *p < 0.05, **p < 0.01, ***p < 0.001. **B** Circulating RIPK3 levels showed a good correlation with markers of cell death such as K18 and cK18 as well as markers of inflammation such as sCD163, IL6, IL8, IL1ra Markers were taken from previous analyses [K18 and cK18—MacDonald S et al. [9]; sCD163, IL6, IL8, IL1ra—Claria J et al. [5]] on the CANONIC cohort. Correlations were calculated as Pearson correlation coefficient.

In patients with acute decompensation (*n* = 311) drawn from the derivation cohort, 187 (60.1%) patients had no ACLF and 124 (39.9%) had ACLF at enrollment. At 28 days and 90 days, 31 (12.4%) and 57 (22.7%) of the total number of patients had died respectively. Fifty-two patients (27.8%) who presented with no ACLF progressed to ACLF following admission (Fig. S1). The median RIPK3 levels were raised in a step-wise manner from 6248 pg/mL (interquartile range [IQR], 3760–9773) in AD, to 10,200 pg/mL (IQR, 6858–17152) in ACLF grade 1, and finally 20,981 pg/mL (IQR, 9296–38,877) in ACLF grade 2/3 (*p* < 0.001 for overall comparison) (Table 2, Fig. 1A).

**Table 2:** Biomarkers stratified by patient group according to the PIRO concept (baseline—derivation cohort).

	RIPK3 (pg/ml) Median (IQR)	*p* value
Disease severity		
Healthy controls (*n* = 21)	**322 (136–493)**	
Stable cirrhosis (*n* = 42)	**445 (320–565)**	
All decompensated (*n* = 311)	**7816 (4500–140,905)**	**<0.001**
No ACLF (*n* = 186)	**6248 (3760–9773)**	
ACLF grade 1 (*n* = 66)	**10,200 (6858–17,152)**	
ACLF grade 2 or 3 (*n* = 58)	**20,981 (9296–38,877)**	**<0.001**
AD throughout (*n* = 134)	**5692 (3331–8436)**	
AD to ACLF (*n* = 52)	**8046 (5161–13,482)**	**0.001**
Survival		
28-day survivor (*n* = 220)	**7215 (4077–12,141)**	
28-day non-survivor (*n* = 31)	**15,761 (9025–39,237)**	**<0.001**
90-day survivor (*n* = 194)	**7080 (3982–11,678)**	
90-day non-survivor (*n* = 57)	**12,104 (6135–26,039)**	**<0.001**
Epidemiological factors		
Age <50 (*n* = 70)	**12,016 (5534–20,758)**	
Age ≥50 (*n* = 224)	**7231 (4307–13,228)**	**0.006**
Male (*n* = 214)	7878 (4665–14,928)	
Female (*n* = 97)	7681 (4225–14,765)	0.983
Etiology		
Etiology no alcohol (*n* = 94)	**6946 (3965–11,075)**	
Etiology alcohol (*n* = 156)	**9119 (5135–14,802)**	**0.027**
Etiology no HCV (*n* = 163)	8037 (4670–14,296)	
Etiology HCV (*n* = 75)	7816 (3928–14,121)	0.554
Etiology no NASH (*n* = 220)	8014 (4307–14,177)	
Etiology NASH (*n* = 17)	6421 (4434–14,739)	0.360
Etiology no others (*n* = 217)	8037 (4548–14,042)	
Etiology others (*n* = 21)	6421 (3076–16,364)	0.360
Disease complication		
No ascites (*n* = 77)	**7816 (3787–14,404)**	
Ascites (*n* = 144)	**10,742 (6351–19,311)**	**0.003**
Injury		
No infection (*n* = 217)	**7241 (4137–13,217)**	
Infection (*n* = 74)	**10,764 (5699–20,743)**	**0.002**
No active alcoholism (*n* = 229)	**7189 (4225–12,971)**	
Active alcoholism (*n* = 46)	**16,554 (8088–31,459)**	**<0.001**
No GI bleeding (*n* = 215)	7990 (5014–13,273)	
GI bleeding(*n* = 36)	7279 (3635–14,758)	0.560
Response		
White blood cell <8 (× 10^9^/L) (*n* = 175)	**6525 (3848–10,267)**	
White blood cell 8–12 (× 10^9^/L) (*n* = 61)	**10,232 (5470–17,842)**	
White blood cell >12 (× 10^9^/L) (*n* = 75)	**11,551 (6583–29,985)**	**<0.001**

*ACLF* acute on chronic liver failure, *AD* acute decompensation, *cK18* caspase-cleaved keratin 18, *K18* keratin 18, *IQR* interquartile range, *RIPK3* receptor interacting protein kinase 3.

**Fig. 2:**
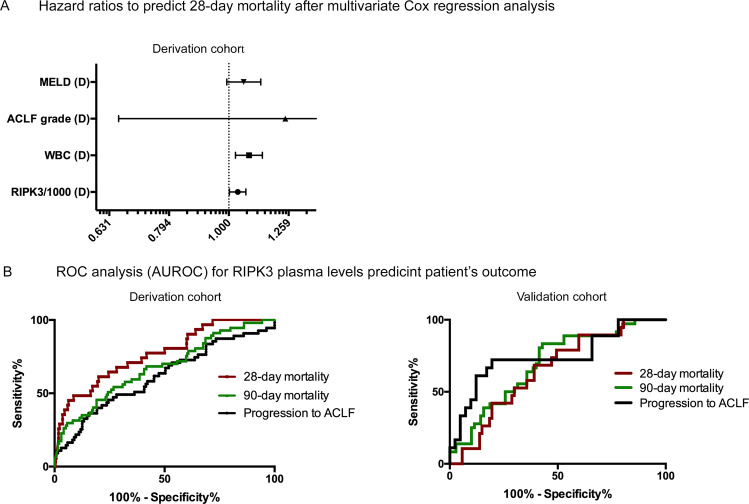
Multivariate COX-regression and ROC analysis. These figures delineate the prognostic relevance of RIPK3 levels. **A** Multivariate Cox-regression analysis in 311 patients with acute decompensation (derivation cohort) considered age, MELD score, WBC, ACLF grade and RIPK3 levels as confounders for 28-day mortality. RIPK3 levels (HR 1.035; 95% CI 1.002–1.068; *p* = 0.037) and WBC (HR 1.081; 95% CI 1-026 1.026–1.138; *p* = 0.003) remained as independent predictors. **B** Thereafter, the predictive power (AUROC) of RIPK3 to predict 28-day and 90-day mortality and progression to ACLF was calculated by ROC analysis in the derivation and validation cohorts. The AUROC of RIPK3 to predict progression to ACLF was 0.650 (95% CI 0.562–0.738) in the derivation cohort and 0.744 (95% CI 0.595–0.892) in the validation cohort. The discrimination power (AUROC) of RIPK3 to predict survival was 0.768 (95% CI, 0.679–0.858) and 0.676 (95% CI, 0.595–0.758) at 28 and 90 days in the derivation cohort and 0.653 (95% CI, 0.530–0.776) and 0.696 (95% CI, 0.593–0.799) in the validation cohort, respectively.

Patients who presented with AD and then developed ACLF during hospitalization had significantly higher plasma RIPK3 levels compared with those who did not progress (*p* = 0.001) (Table 2, Fig. 1A). The area under the receiver-operating curve (AUROC) of RIPK3 to predict progression to ACLF was 0.650 (95% confidence interval [CI], 0.562–0.738) (Table S6, Fig. 2). In addition, we observed that RIPK3 plasma levels were statistically higher in non-survivors compared with survivors at 28-days (*p* < 0.001) and 90-days (*p* < 0.001) (Table 2, Fig. 1A). The discrimination power (AUROC) of RIPK3 to predict survival was 0.768 (95% CI, 0.679–0.858) and 0.676 (95% CI, 0.595–0.758) at 28 and 90 days, respectively (Table S6, Fig. 2B).

RIPK3 levels were significantly higher in those with alcohol-related cirrhosis and also in patients with infections and higher white blood cell count (WBC) (Table 2, Fig. 1A). Patients with ascites and/or younger patients (>50 years) also had more elevated RIPK3 levels (Table 2, Fig. 1A). RIPK3 were also higher in the presence of any type of organ failure apart from respiratory failure and more importantly increased in patients with single liver failure compared to no organ failure (Table 2, Fig. 1A). However, in the derivation cohort, plasma RIPK3 was also elevated in patients with single kidney failure but the levels were lower than those with single liver failure. Not surprisingly, in those with multiple organ failures, there was an elevation in RIPK3 (Table 2, Fig. 1A) and markers of inflammation and liver function (Table 2, Fig. 1A).

Circulating RIPK3 levels correlated well with other markers of cell death such as caspase-cleaved keratin 18 (cK18) (apotototic cell death) and K18 (total cell death, including apoptosis) as well as inflammatory such as sCD163, interleukin (IL)−6, IL-8, and IL-1ra (Fig. 1B). Multivariate Cox regression analysis correcting for age, gender, MELD score, WBC and ACLF grade, revealed RIPK3 levels (hazard ratio [HR], 1.035; 95% CI, 1.002–1.068]; *p* = 0.037) and WBC (HR, 1.081; 95% CI [1.026–1.138]; *p* = 0.003) as independent predictors for 28-day mortality (Table S7, Fig. 2A).

#### Validation cohort

In patients with acute decompensation from the validation cohort, 59 (55.7%) patients had no ACLF and 47 (44.3%) had ACLF at enrollment. At 28 days and 90 days, 19 (17.9%) and 39 (36.8%) of the total number of patients had died respectively. Eighteen patients (30.5%) who presented with no ACLF progressed to ACLF following admission (Fig. S1B). In this cohort, we confirmed our previous observation that ACLF is associated with elevated levels of plasma markers of RIPK3 in comparison to no ACLF (Tables S8 and S9, Fig. S4). The discrimination power (AUROC) of RIPK3 to predict survival was 0.653 (95% CI, 0.530–0.776) and 0.696 (95% CI, 0.593–0.799) at 28 and 90 days, respectively (Table S6, Fig. 2B).

Additional markers of cell death were also increased in patients with ACLF. In particular, levels of cK18, K18 and circulating nucleosomes were significantly raised in a step-wise manner with increasing ACLF grade (Tables S8 and S9). Interestingly, as K18 increased more steeply than the cK18 with increasing clinical severity, the cK18:K18 ratio also dropped [from 0.89 (0.58–2.50) in no ACLF, to 0.71 (0.50–2.44) in ACLF 1, and to 0.53 (0.34–1.91) in ACLF 2/3 (*p* = 0.081), respectively] suggesting, as previously reported, that non-apoptotic rather than apoptotic modes of cell death are relatively more important in the pathophysiology of ACLF (Tables S8 and S9).

### Immunohistochemistry: RIPK1, RIPK3, and pMLKL

**Fig. 3:**
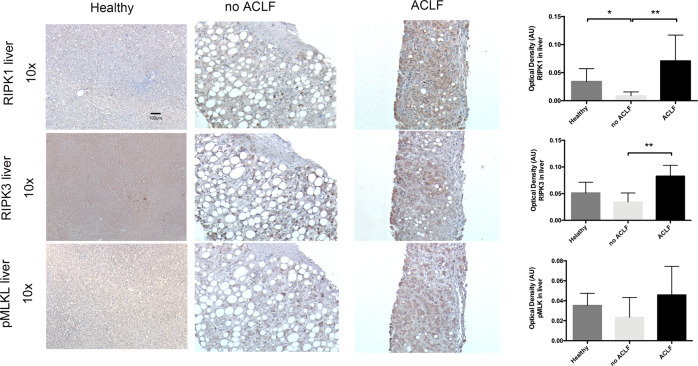
Immunohistochemistry human liver tissue. Representative images of liver biopsies of patients with alcoholic hepatitis with and without ACLF stained for RIPK1, RIPK3, and pMLKL showing increased staining in the ACLF patients. Group comparisons were performed by Mann–Whitney U test. *p < 0.05, **p < 0.01, ***p < 0.001.

In order to determine whether the circulating RIPK3 was originating from the liver and reflected hepatic necroptosis, biopsies from patients with AD with and without ACLF were stained for RIPK1, RIPK3, and phosphorylated MLKL (pMLKL) (Table S4, Fig. 3). Although RIPK1, RIPK3, and pMLKL were expressed in biopsies of AD patients, this was considerably upregulated in patients with ACLF and additional immuno-staining confirmed low expression of cleaved caspase 3 and 8 (Fig. S5). The presence of positive staining of all key components of the necroptotic pathway and in particular the presence of the necrosome-phosphorylated form of MLKL responsible for cellular death, suggests that hepatic necroptosis is an important feature of human ACLF.

### The rodent model of ACLF recapitulates RIPK1 associated cell death in human ACLF

In keeping with our previous studies [9], rodents who had undergone bile-duct ligation (BDL, model of advanced fibrosis) demonstrated significant alterations in liver function tests compared with sham-operated controls (Table S10) but did not demonstrate significant elevation in circulating nucleosomes or RIPK3 when compared to sham-operated animals (Fig. S6). Immunohistochemistry of liver and kidney tissues from BDL animals demonstrated positive staining for RIPK1 and RIPK3 in comparison to sham animals (Fig. S6). Induction of ACLF in BDL animals by administration of LPS led to significant worsening of liver function tests [alanine aminotransferase (ALT), 79.4 ± 12.8 IU/L (mean ± SD); aspartate transaminase (AST), 412.9 ± 39.4 IU/L; *p* = 0.031 compared to BDL] mirrored by increased circulating nucleosomes. In the BDL + LPS animals, a dramatic significant increase in plasma RIPK3 levels was also observed compared to BDL (*p* = 0.042) and this was accompanied by increased protein expression of both RIPK1 and RIPK3 in liver and kidney tissues (Fig. S6). These data strongly demonstrate that the onset of ACLF triggered by LPS administration to the BDL animals is associated with initiation of necroptosis within 3 h, confirming the findings in the ACLF patients.

### LPS induces a switch from caspase-dependent cell death to RIPK1 mediated cell death

**Fig. 4:**
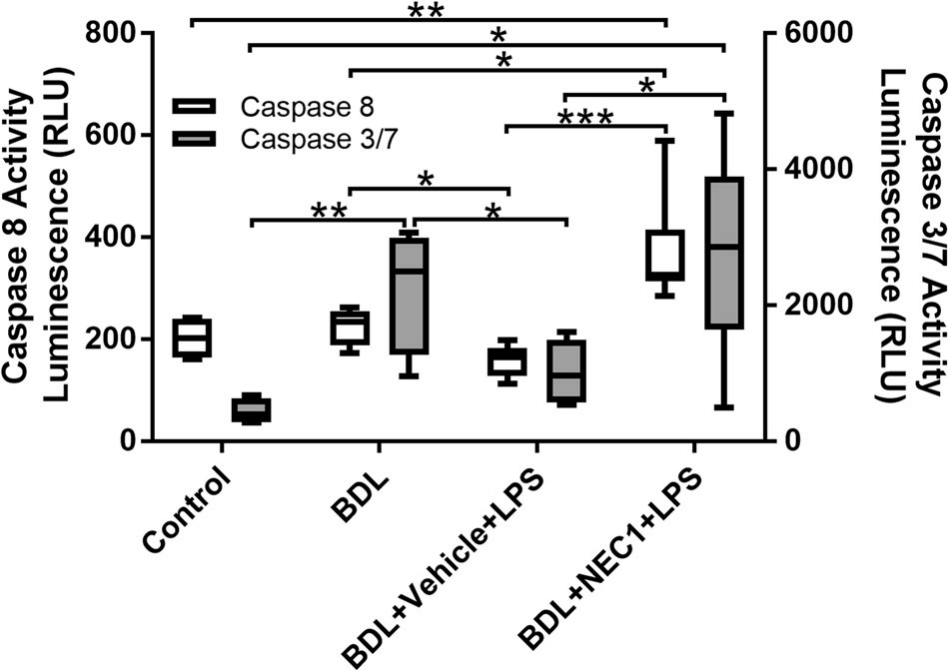
Caspase activity liver tissue. LPS induces a switch from Caspase-dependent cell death to necroptosis. Caspase 8 and 3/7 activities in liver tissue of sham, BDL, BDL + LPS and BDL + NEC-1 + LPS. Data are presented as box and whisker plots. Data were analyzed using Student’s *t* test or Mann–Whitney U test. *p < 0.05, **p < 0.01, ***p < 0.001.

To further explore the switch from predominantly apoptotic cell-death in cirrhosis to necroptosis in ACLF, we evaluated the relative liver Caspases-3/7 and 8 activities in the animals described above. Caspase-8 is a key regulator of the two forms of cell death (apoptosis and necroptosis). Activation of caspase-8 (initiator caspase) promotes apoptosis through caspases-3/7 (executioner caspases). On the other hand, suppression of caspase-8 activity shifts the balance towards necroptosis. Therefore, we investigated the activation pattern of both caspase-8 and caspase-3/7 in liver homogenates. BDL livers showed a significant increase in caspase-3/7 activation compared to control, with a median activation of 2492 RLU (IQR, 1273–2988) and 396 RLU (IQR, 279–630), respectively (*p* = 0.0087). In contrast, caspase-8 activation did not differ between the two groups with a median activation of 234.5 (IQR, 187.5–255.3) and 202 (IQR, 163.8–239.5), respectively (*p* = 0.425). In the “BDL + Vehicle+LPS” group a significant reduction of all caspase activities was observed compared to BDL (caspase-3/7 [RLU] median 968 RLU (IQR 571-1495), *p* = 0.035 compared to BDL; caspase-8, median 164 RLU (IQR 128-182.5), *p* = 0.021 compared to BDL]. NEC-1, inhibiting RIPK1-dependent necroptosis, restored an apoptotic phenotype as demonstrated by caspases-3/7 and caspase-8 activation, similar to that observed in the BDL group without LPS [caspase-3/7, median 2857 RLU (IQR 1640-3890); caspase-8, median 326.5 (IQR 313.5-414.3), *p* = 0.626 and *p* = 0.020, compared to BDL, respectively] (Fig. 4).

### NEC-1 treatment prevents the occurrence of ACLF in the rodent ACLF model

As increased liver expression of both RIPK1 and RIPK3 and elevated levels of TNFα [31–33] are associated with onset of the ACLF phenotype in both humans and rodents, a canonical TNF-mediated RIPK1-RIPK3-dependent necroptosis might account for increased cell death and organ failure in ACLF. To determine whether necroptosis is causally related to the pathogenesis of ACLF, we explored whether treatment with NEC-1would prevent ACLF development.

**Fig. 5:**
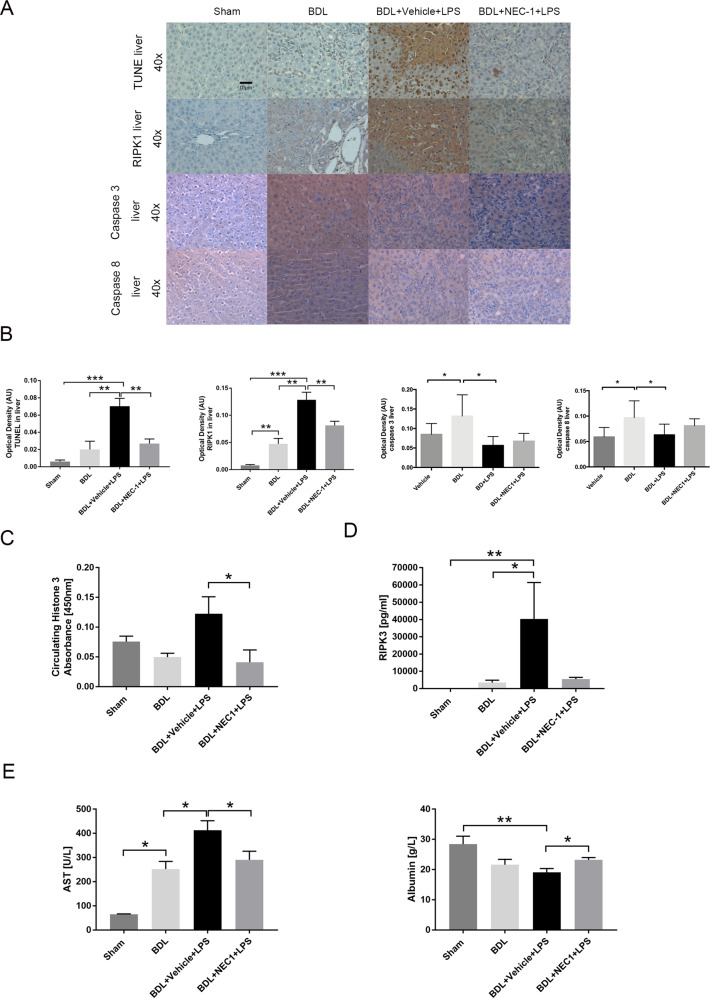
Effect of RIPK1 inhibition by Necrostatin 1. Histological assessment and biomarkers show the protective effect of pharmacological RIPK1 inhibition against ACLF-related liver injury. **A** Representative images of liver tissue of sham, BDL, BDL + LPS and BDL + NEC-1 + LPS stained for TUNEL, RIPK1, cleaved-Caspase 3 and 8. **B** The optical density of immunostaining intensity (mean ± SEM) was assessed by Image J (mean ± SEM). **D** Plasma levels of AST and albumin of sham, BDL, BDL + LPS, and BDL + NEC-1 + LPS (mean ± SEM). **C**–**E** Plasma levels of ALT, albumin, RIPK3 and histone 3 of sham, BDL, BDL + LPS and BDL + NEC-1 + LPS (mean ± SEM). Data were analyzed using Student’s *t* test or Mann–Whitney U test. *p < 0.05, **p < 0.01, ***p < 0.001.

NEC-1 treatment significantly decreased the expression of RIPK1 in NEC-1-treated “BDL + LPS” group compared to the “BDL + vehicle+LPS” group (*p* = 0.009) (Fig. 5A). Moreover, TUNEL staining showed a significant reduction in total cell death in the livers of the BDL + NEC-1+LPS compared to the BDL + Vehicle+LPS group (*p* = 0.001) (Fig. 5B), mirrored by a significant reduction in circulating histone 3 levels in the NEC-1 treated group, compared to BDL + Vehicle+LPS animals (*p* = 0.048) (Fig. 5C). Immunohistochemistry on liver tissue after BDL + Vehicle+LPS showed a reduction of cleaved caspase 3 and 8 after LPS injection (*p* = 0.05 for both), consistent with the reduced activities shown above, and tended to increase after NEC-1 treatment (*p* < 0.001 for both) (Fig. 5A, B) which mirrors the enzyme activity shown on Fig. 4. In order to further evaluate whether inhibition of RIPK1 could account for the observed attenuation of total cell death, we measured circulating levels of RIPK3. Plasma RIPK3 levels in the BDL + NEC-1+LPS group reduced markedly compared to the BDL + Vehicle+LPS (*p* = 0.073) (Fig. 5D). Furthermore, liver preservation by NEC-1 was also demonstrated by significantly lower values of AST (*p* = 0.037) and a significantly higher albumin levels (*p* = 0.017) (Fig. 5E).

We then evaluated whether the preservation of liver function induced by NEC-1 pre-treatment was accompanied by amelioration of kidney and brain dysfunction. TUNEL staining showed a significant reduction in cell death in kidney sections of BDL + NEC-1+LPS group compared to BDL + vehicle+LPS (*p* = 0.002) (Fig. S7), which was associated with a significant reduction in creatinine (*p* = 0.020) and urea levels (*p* = 0.012) in BDL + NEC-1+LPS compared to BDL + Vehicle+LPS (Fig. S7), demonstrating that prevention of necroptosis by NEC-1 treatment protects also from kidney injury. Furthermore, a reduction in brain water content, although not statistically significant, was observed in the BDL + NEC-1+LPS treated group compared to BDL + Vehicle+LPS (*p* = 0.100) (Fig. S7).

### RIPK1 inhibition by RIPA56 prevents RIPK1/RIPK3 mediated necroptosis in a CCl4-GalN mouse model

**Fig. 6:**
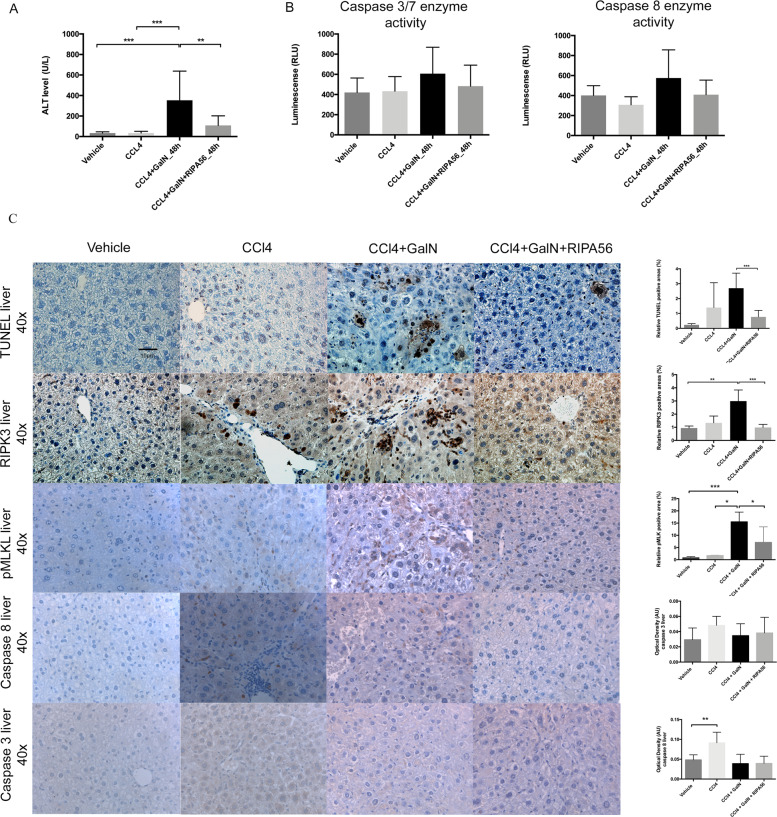
Effect of RIPK1 inhibition by RIPA56 in a murine model of ACLF. C57BL/6 mice were treated with CCl_4_ for 6 weeks and subsequently injected with GalN. All animals were sacrificed 48 h after GalN injection. **A** GalN injection was associated with a significant increase of ALT levels. **B** Caspase 3/7 and 8 expression showed a mixed response without proving significance. **C** TUNEL staining of liver tissue showed that GalN injection induced hepatocyte cell death with high expression of RIPK3 (magnification, 10x and 40x). RIPK1 inhibition by RIPA 56 significantly reduced ALT levels, abrogated tissue injury (TUNEL staining) and reduced the liver RIPK3, pMLK, cleaved-Caspase 3 and 8 expression. The optical density of immunostaining intensity (mean ± SEM) was measured. Data were analyzed using Student’s *t* test or Mann–Whitney U test. Only significant *p* values were displayed *p < 0.05, **p < 0.01, ***p < 0.001.

In order to validate the results of the experiments in the BDL animals, we used a second model of ACLF and a more specific inhibitor of RIPK1, RIPA56. In this mouse model, the chronic liver injury was induced by 6-weeks carbon tetrachloride (CCl_4_) gavage followed by Galactosamine (GalN) injection as the second hit. This model was designed to simulate ACLF that occurs due to a direct liver injury (unlike LPS) and produces predominant liver failure. ALT levels increased from 64.8 ± 54.2 U/L in CCl_4_ only treated animals to 353.5 ± 284.2 U/L in the CCl_4_-GalN animals (*p* < 0.001) (Fig. 6A) and TUNEL staining of liver tissue demonstrated a significant hepatocyte cell death after injection of GalN (*p* < 0.001) (Fig. 6B). RIPK3 immunohistochemistry showed that hepatocyte cell death was associated with TUNEL positivity, increased RIPK3 (*p* < 0.01) and pMLK expression (*p* < 0.01) and decreased caspase 3 and 8 expression (Fig. 6B) principally defining a necroptotic cell death in this model. RIPK1 inhibition by RIPA56 (selectivity shown by Ren Y et al. [26]) significantly abrogated the tissue injury (ALT level *p* = 0.006, TUNEL stain *p* < 0.001) and liver RIPK3 (*p* < 0.001) and pMLK expression (*p* < 0.01) suggesting that ACLF is associated with RIPK1/RIPK3 mediated necroptotic cell death in the liver (Fig. 6B).

## Discussion

This study describes for the first time that plasma RIPK3 levels are associated with the progression of patients with no ACLF to ACLF, severity of ACLF and mortality in two separate clinical cohorts suggesting that RIPK1 mediated cell death is an important mechanism of cell death in the patients with ACLF. This was associated with markedly increased expression of RIPK1, RIPK3, and pMLKL in liver tissues confirming the importance of this pathway in mediating hepatocyte injury in these patients. The important role of RIPK1 mediated cell death in ACLF was demonstrated in two animal models of ACLF. Administration of two separate inhibitors of RIPK1, NEC-1 or RIPA56, resulted in the prevention of development of ACLF in the animal models. Taken together these data point to the importance of necroptosis as being important in the pathogenesis of ACLF.

We have previously reported the importance of onset of non-apoptotic cell death in patients with ACLF [9]. The significant increase in the circulating levels of RIPK3 in patients with ACLF supports the notion that RIPK1 mediated cell death is likely to be the operative mechanism. RIPK3 is a key component in the necroptotic pathway alongside RIPK1 and pMLKL. It is also associated with mitochondrial energy metabolism and production of excess reactive oxygen species [20]. Circulating RIPK3 levels not only reflected the severity of ACLF in the present study but also identified patients likely to progress to develop ACLF and patients likely to die at 28-day and 90-day. These data were validated in a second cohort although the AUROC for 28-day mortality was lower possibly due to the small sample size and different timing of sampling. Importantly RIPK3 levels correlated closely with markers of systemic inflammation, which have been shown to be the central in the mechanism underlying the development of ACLF [31]. In order to determine whether this was simply an association or whether RIPK1 mediated cell death was pathophysiologically important in the development of ACLF, we designed a study to determine whether inhibition of the key upstream necroptosis component RIPK1 using NEC-1 and RIPA56 would prevent the development of ACLF in rodent models.

The most important observation in this study was that NEC-1, an inhibitor of RIPK1, prevented hepatic and renal cell death in a BDL model of ACLF induced by injection of LPS, providing the first proof of the importance of this pathway in the pathogenesis of ACLF from AD. This model, extensively validated previously, mimics the clinical situation where ACLF is precipitated by bacterial infection and exhibits cerebral, circulatory and renal failure, all important extrahepatic diagnostic features of ACLF. The observed over-expression of RIPK1 in the liver and kidney in the animal model of ACLF was associated with evidence of cell death. It is intriguing to note that renal tubular injury was also prevented with NEC-1 in this model suggesting that necroptosis may also be the operative mechanism of kidney injury. In order to determine whether the RIPK1 mediated cell death was also important in a ‘hepatic’ form of ACLF where liver injury predominates, a new model of CCL_4_-GalN was developed. Interestingly, this model develops severe liver injury but no extrahepatic organ failures, such as kidney and brain failure. In this second model RIPA56, another inhibitor of RIPK1, was used as recently described to be more selective, potent and with no off-target effect compared to NEC-1, and its administration also abrogated severity of liver injury significantly [14]. Taken together, the data suggest that necroptosis is likely to be pathophysiologically important in the pathogenesis of ACLF and RIPK1 is a potential therapeutic target.

Normalizing RIPK1 is known to modulate the NF-κβ signaling pathway [34], which can promote cell survival thereby preventing development of ACLF [35]. An alternative mechanism of the protective effect of inhibition of RIPK1 may by through modulation of necroptosis that is triggered in BDL animals by induction of senescence [36–38]. In addition, we showed that Caspase 8 activity in ACLF rodent model (BDL + LPS) was suppressed suggesting a possible switch from apoptosis to necroptosis. Interestingly, NEC-1 prevented loss of Caspase 8 activity and onset of LPS-induced necroptosis. However, whether these observations on the potential role of necroptosis in ACLF can be translated into clinical application as a therapeutic, needs further evaluation as targeting RIPK3-mediated necroptosis was effective in preventing APAP induced acute liver failure in some studies [23, 39, 40] but not in others [41]. Of concern, was the observation that in concanavalin A (ConA)-induced autoimmune hepatitis, RIPK3 deletion was protective, whereas RIPK1 inhibition exacerbated disease, accelerated animal death, and was associated with increased hepatocyte cell death [40]. However, other data suggested that pre-treatment with NEC-1 had protective effect against ConA mediated injury [42–44]. The mechanism underlying these variable effects are unclear but may be related to the stage of the disease and potential activation of alternative pathways, particularly in the animals with RIPK3 deletion.

Circulating nucleosomes, a DAMP [45], also reflected the severity of ACLF. In addition, our study showed that circulating nucleosomes were associated with brain, heart, and respiratory failure. Histones, components of nucleosomes released into the circulation from damaged cells, are known to promote inflammation [46]. Our results are in concert with the finding that circulating nucleosomes are also elevated in other clinical conditions associated with necrosis such as severe acute pancreatitis [47] and circulating histones are elevated in acute liver failure [46]. Histones released after tissue trauma may lead to multiple organ injury and death [48]. Several studies suggest that histones are both biomarkers of disease progression and also possible therapeutic targets in sepsis, acute liver failure, and other inflammatory diseases [46–49]. In the rodent model of ACLF, NEC-1 also prevented the LPS-induced liver damage and the consequent release of histone-3, which may have partially contributed to its protective effect.

Acute kidney injury is an important feature of ACLF [50] and tubular cell death is an important pathophysiological characteristic of this syndrome [51]. In the present study, we also showed that renal dysfunction was associated with tubular injury. Interestingly, the expression of RIPK1 and RIPK3 in the kidney of the animal model of ACLF was significantly increased. Previous studies have also reported that plasma RIPK3 levels are elevated in patients with AKI following trauma [52]. Pharmacological inhibition of RIPK1 with NEC-1 protected against renal tubular cell death in our BDL model of ACLF suggesting that AKI in ACLF is due to renal tubular necroptosis. Alternatively, or in addition, AKI in ACLF may be consequent on release of DAMPs from liver injury. The study also showed that the brain water of the BDL animals with ACLF was markedly reduced in the NEC-1 treated group and this was independent of changes in the ammonia levels. The mechanism of protection of the brain by NEC-1 is not clear but may be consequent on protection of liver cell death and DAMP release.

There are some limitations of our study. Firstly, the mechanism and source of the circulating RIPK3 is unclear. However, when comparing no organ failure and single organ failure, we observed that the elevated plasma RIPK3 levels were associated with single liver failure. In addition, histological assessment showed high expression of RIPK3 in the liver of ACLF patients and also in the animal model. The elevation of plasma RIPK3 is therefore likely to be predominantly derived from the liver but could also be originating from the kidney as evidence of necroptosis was also observed in the rodent kidneys and the levels were also found to be elevated in the patients with single kidney failure in the patients in the CANONIC study. Secondly, NEC-1 has a short half-life of about 1 h [53] and is known to have off-target effects, such as inhibition of indoleamne-2,3-dioxygenase (IDO), which can modulate immune function [54] and this additional feature may have partially contributed to its observed effects. However, administration of RIPA56, a specific inhibitor of RIPK1 with no anti-IDO activity, had a similar protective effect suggesting that necroptosis is indeed important in the development of ACLF and a potential therapeutic target. We considered using a RIPK3 deletion mouse but the fact that these animals develop variable degrees of liver injury and fibrosis and makes it an unsuitable model of cirrhosis and ACLF [16].

In conclusion, the novel observation that inhibition of RIPK1 with NEC-1 and RIPA56 resulted in prevention of occurrence of ACLF in the two animal models suggests that necroptosis is likely to be of pathogenic importance and a potential therapeutic target to prevent the development of ACLF. This observation is supported by data from two patient cohorts in whom elevated circulating levels of RIPK3 was associated with progression of patients from AD to ACLF, severity of ACLF, and short-term mortality. Taken together, the study suggests that circulating RIPK3 may serve as a potential biomarker to select patients for therapy with drugs targeting RIPK1 mediated cell death.

## Supplementary information

Reproducibility checklist

Supplementary Material

Figure S1

Figure S2

Figure S3

Figure S4

Figure S5

Figure S6

Figure S7

## Data Availability

All data is available on request from the corresponding author.
