# Correction: The role of RIPK1 mediated cell death in acute on chronic liver failure

**DOI:** 10.1038/s41419-022-04874-x

**Published:** 2022-04-29

**Authors:** Takayuki Kondo, Stewart Macdonald, Cornelius Engelmann, Abeba Habtesion, Jane Macnaughtan, Gautam Mehta, Rajeshwar P. Mookerjee, Nathan Davies, Marco Pavesi, Richard Moreau, Paolo Angeli, Vicente Arroyo, Fausto Andreola, Rajiv Jalan

**Affiliations:** 1 Liver Failure Group, Institute for Liver and Digestive Health, University College London, London, UK; 2 Department of Gastroenterology, Graduate School of Medicine, Chiba University, Chiba, Japan; 3 Section Hepatology, Clinic for Gastroenterology and Rheumatology, University Hospital Leipzig, Leipzig, Germany; 4 Department of Hepatology and Gastroenterology, Campus Virchow-Klinikum and Charité Campus Mitte, Charité - Universitaetsmedizin Berlin, Berlin, Germany; 5 The Roger Williams Institute of Hepatology, Foundation for Liver Research, London, UK; 6 European Foundation of the study of Chronic Liver Failure (EF-CLIF), Barcelona, Spain; 7 Inserm, U1149, Centre de Recherche sur l’Inflammation (CRI), Clichy, Paris, France; 8 UMRS1149, Université de Paris, Paris, France; 9 Assistance Publique-Hôpitaux de Paris, Service d’Hépatologie, Hôpital Beaujon, Clichy, France; 10 Unit of Internal Medicine and Hepatology (UIMH), Department of Medicine - DIMED University of Padova, Padova, Italy

**Keywords:** Hepatitis, Liver diseases

Correction to: *Cell Death and Disease* 10.1038/s41419-021-04442-9, published online 17 December 2021

The original version of this article unfortunately contained a mistake. For the author Gautam Mehta one affiliation was omitted. The omitted affiliation is: The Roger Williams Institute of Hepatology, Foundation for Liver Research, London, UK. The original article has been corrected.

