# Supplementary material for: The role of RIPK1 mediated cell death in acute on chronic liver failure
Source: Cell Death Dis. 2021 Dec 17;13(1):5. doi: 10.1038/s41419-021-04442-9 (PMC8683430; doi:10.1038/s41419-021-04442-9)
Supplement: Supplementary file 2 — Supplementary Material [file 41419_2021_4442_MOESM2_ESM.docx]

**The role of RIPK1 mediated cell death in acute on chronic liver failure**

Takayuki Kondo^1,2^, Stewart Macdonald^1^, Cornelius Engelmann^1,3,4^, Abeba Habtesion^1^, Jane Macnaughtan^1^, Gautam Mehta^1^, Rajeshwar P Mookerjee^1^, Nathan Davies^1^, Marco Pavesi^5^, Richard Moreau^5,6^, Paolo Angeli^5,7^, Vicente Arroyo^5^, Fausto Andreola^1^, Rajiv Jalan^1*^

^1^ Liver Failure Group, Institute for Liver and Digestive Health, University College London, London, United Kingdom.

^2^ Department of Gastroenterology, Graduate School of Medicine, Chiba University, Chiba, Japan.

^3^ Section Hepatology, Clinic for Gastroenterology and Rheumatology, University Hospital Leipzig, Leipzig, Germany

^4^ Department of Hepatology and Gastroenterology, Campus Virchow-Klinikum and Charité Campus Mitte, Charité - Universitaetsmedizin Berlin, Berlin, Germany

^5^ European Foundation of the study of Chronic Liver Failure (EF-CLIF), Barcelona, Spain

^6^ Inserm, U1149, Centre de Recherche sur l'Inflammation (CRI), Clichy and Paris, France ; UMRS1149, Université de Paris, Paris, France ; Assistance Publique-Hôpitaux de Paris, Service d'Hépatologie, Hôpital Beaujon, Clichy, all in France

^7^ Unit of Internal Medicine and Hepatology (UIMH), Department of Medicine - DIMED University of Padova, Padova, Italy

Dr Kondo, Dr Macdonald and Dr Engelmann are joint 1^st^ authors

Dr Andreola and Prof Jalan are joint senior authors

**Table of content**

Supplementary figures – Page 2 - 3

Supplementary tables – Page 4 - 13

**Supplementary figure legends:**

Figure S1: Patient flow diagram

Consort diagram of patient flow of the A) CANONIC (derivation) cohort and B) DASIMAR (validation) cohort

Figure S2: Negative and isotype controls for immunohistochemistry

Figure S3: RIPK3 antibody validation with blocking peptide

Representative images of liver tissues of rat models of sham and BDL + LPS stained for RIPK3 with or without blocking peptide.

Figure S4: RIPK3 plasma levels in different patients subgroups

Patients with more severe end-stage liver cirrhosis had higher circulating RIPK3 levels. Furthermore, RIPK3 levels were higher in single liver failure compared to single renal failure suggesting the liver as the origin for blood RIPK3 levels. Non-surivors, patients with high WBC (>12 exp9/L) and patients with alcoholic liver disease (ALD) also had higher circulating RIPK3 levels.

Figure S5: Immunohistochemistry human liver tissue for cleaved Caspase 3 and 8

Representative images of liver biopsies of patients with alcoholic hepatitis with and without ACLF stained for cleaved caspase 3 and 8 showing no significantly increased staining in the ACLF patients.

Figure S6: BDL + LPS ACLF rat model

Rat model of ACLF display increased protein expression of RIPK1 and RIPK3 in liver and kidney. (A) Plasma levels of RIPK3 and nucleosomes of rat models of sham, BDL and BDL + LPS (mean ± SEM). (B) Representative images of liver and kidney tissues of rat models of sham, BDL and BDL + LPS stained for RIPK1 and RIPK3 (magnification, 10x and 40x). (C) The optical density of RIPK1 and RIPK3 immunostaining intensity (mean ± SEM). Data were analyzed using Student’s *t* test or Mann-Whitney U test. **p*<0.05, ***p*<0.01, ****p*<0.001.

Figure S7: Necrostatin 1 in a BDL + LPS ACLF rat model

Pharmacological RIPK1 inhibition protects against ACLF-related multiple organ injury. (A) Representative images of kidney tissue of sham, BDL, BDL + LPS and BDL + NEC-1 + LPS of TUNEL staining (magnification, 10x and 40x). The optical density of TUNEL staining intensity (mean ± SEM). (B) Plasma levels of creatinine and urea of sham, BDL, BDL + LPS and BDL + NEC-1 + LPS (mean ± SEM). (C) Brain water of BDL + LPS and BDL + NEC-1 + LPS (mean ± SEM). Data were analyzed using Student’s t test or Mann-Whitney U test. **p*<0.05, ***p*<0.01, ****p*<0.001

Supplementary tables

**Table S1: Patient characteristics stratified by survivors or non-survivors at 28-day and 90-day – derivation cohort**

|  | 28-day | | | 90-day | | |
| --- | --- | --- | --- | --- | --- | --- |
|  | Survivors  (n=220) | Non-survivors  (n=31) | *p*  values | Survivors  (n=194) | Non-survivors  (n=57) | *p*  values |
| Age (years) | 58 (22-86) | 57 (43-77) | 0.683 | 58 (22-68) | 56 (37-81) | 0.675 |
| Male (n, %) | 156 (70.9) | 20 (64.5) | 0.530 | 142 (72.2) | 34 (59.6) | 0.069 |
| *Etiology (n, %)* |  |  |  |  |  |  |
| Alcohol | 136 (62.1) | 20 (64.5) | 0.846 | 119 (61.3) | 37 (66.1) | 0.639 |
| Alcohol + HCV | 100 (48.3) | 19 (63.3) | 0.364 | 85 (46.4) | 34 (63) | 0.192 |
| HCV | 70 (33.7) | 5 (16.7) | 0.091 | 63 (34.4) | 12 (21.8) | 0.098 |
| Non-alcoholic steatohepatitis | 14 (6.8) | 3 (10) | 0.459 | 11 (6) | 6 (10.9) | 0.237 |
| Other | 19 (9.1) | 2 (6.9) | >0.999 | 16 (8.7) | 5 (9.3) | 0.999 |
| Active alcoholism at enrollment  (n, %) | 29 (14.2) | 4 (13.8) | >0.999 | 23 (12.8) | 10 (18.5) | 0.372 |
| Ascites (n, %) | **90 (60.4)** | **26 (89.7)** | **0.002** | **72 (56.7)** | **44 (86.3)** | **<0.001** |
| Gastrointestinal bleeding (n, %) | 34 (15.5) | 2 (6.5) | 0.273 | **33 (17)** | **3 (5.3)** | **0.03** |
| Bacterial infection (n, %) | **40 (18.3)** | **14 (45.2)** | **0.002** | **33 (17.1)** | **21 (37.5)** | **0.003** |
| *Organ failure (n, %)* |  |  |  |  |  |  |
| Liver | **29 (13.2)** | **15 (48.4)** | **<0.001** | **22 (11.3)** | **22 (38.6)** | **<0.001** |
| Kidney | **48 (21.8)** | **12 (38.7)** | **0.045** | **39 (20.1)** | **21 (36.8)** | **0.013** |
| Brain | **20 (9.1)** | **9 (29)** | **0.004** | 18 (9.3) | 11 (19.3) | 0.057 |
| Coagulation | **17 (7.7)** | **8 (25.8)** | **0.005** | **15 (7.7)** | **10 (17.5)** | **0.042** |
| Cardiac^1^ | **9 (4.1)** | **5 (16.1)** | **0.019** | 8 (4.1) | 6 (10.5) | 0.094 |
| Respiratory | 4 (1.8) | 2 (6.5) | 0.162 | 4 (2.1) | 2 (3.5) | 0.621 |
| *Laboratory values* |  |  |  |  |  |  |
| White blood cell (x10^9^/L) | **5.9 (0.9-30.5)** | **8.8 (0.9-34.3)** | **<0.001** | **5.7 (0.9-22.9)** | **11.4 (0.9-34.3)** | **<0.001** |
| Bilirubin (mg/dL) | **2.7 (0.3-39.9)** | **10 (0.5-44.8)** | **<0.001** | **2.6 (0.25-39.9)** | **7 (0.5-44.8)** | **<0.001** |
| Prothrombin time  (international normalized ratio) | **1.5 (1-6.9)** | **2 (1.2-4.5)** | **<0.001** | **1.4 (1-6.9)** | **2 (1.1-4.5)** | **<0.001** |
| Albumin (g/dL) | 2.9 ± 0.7 | 2.8 ± 0.8 | 0.239 | 3 ± 0.6 | 2.8 ± 0.8 | 0.233 |
| Creatinine (mg/dL) | 1.1 (0.4-8.2) | 1.4 (0.6-6.4) | 0.15 | **1.1 (0.4-8.2)** | **1.4 (0.5-6.4)** | **0.025** |
| Sodium (mmol/L) | 136 (114-154) | 132 (125-149) | 0.146 | **137 (114-154)** | **131 (117-149)** | **0.001** |
| Platelets (10^9^/L) | 79 (13-480) | 68 (14-372) | 0.568 | 78 (13-480) | 82 (14-372) | 0.852 |
| *Scores* |  |  |  |  |  |  |
| MELD | **18 (7-40)** | **27 (11-40)** | **<0.001** | **18 (7-38)** | **27 (9-40)** | **<0.001** |
| MELD Na | **21 (7-40)** | **30 (11-40)** | **<0.001** | **20 (7-39)** | **30 (11-40)** | **<0.001** |
| Child-Pugh score | **9 (5-15)** | **12 (7-15)** | **<0.001** | **9 (5-15)** | **12 (7-15)** | **<0.001** |
| CLIF-OFs | **7 (6-13)** | **10 (6-17)** | **<0.001** | **7 (6-13)** | **9 (6-17)** | **<0.001** |

Data are expressed as mean ± SD or median (range).

cK18, caspase-cleaved keratin 18; CLIF-OFs, chronic liver failure-organ failure score; HCV, hepatitis C virus; K18, keratin 18; MELD, model for end-stage liver disease; RIPK3, receptor interacting protein kinase 3.

**Table S2: Baseline characteristics stratified by presence of absence or ACLF at enrolment (validation cohort)**

|  | No ACLF (n=59) | ACLF (n=47) | *p* values |
| --- | --- | --- | --- |
| Age (years) | 51 (45-65) | 49 (41-54) | 0.054 |
| Male (n, %) | 32 (54.2) | 30 (63.8) | 0.331 |
| *Etiology (n, %)* |  |  |  |
| Alcohol | **31 (52.5)** | **34 (72.3)** | **0.046** |
| Alcohol + HCV | 8 (13.6) | 3 (6.4) | 0.339 |
| HCV | 5 (8.5) | 2 (4.3) | 0.459 |
| Non-alcoholic steatohepatitis | 6 (10.2) | 2 (2.1) | 0.130 |
| Other | 9 (15.3) | 7 (14.9) | >0.999 |
| Active alcoholism at enrollment (n, %) | 41 (69.5) | 37 (78.7) | 0.376 |
| Ascites (n, %) | 28 (47.5) | 31 (66.0) | 0.077 |
| Gastrointestinal bleeding (n, %) | 17 (28.8) | 10 (21.3) | 0.502 |
| Bacterial infection (n, %) | 19 (32.2) | 15 (31.9) | >0.999 |
| *Organ failure (n, %)* |  |  |  |
| Liver | **6 (10.2)** | **26 (55.3)** | **<0.001** |
| Kidney | - | 25 (53.2) | - |
| Brain | **2 (3.4)** | **9 (19.2)** | **0.011** |
| Coagulation | **2 (3.4)** | **9 (19.2)** | **0.011** |
| Cardiac | 3 (5.1) | 8 (17.0) | 0.058 |
| Respiratory | - | 5 (10.6) | - |
| *Laboratory values* |  |  |  |
| White blood cell (x10^9^/L) | **6.9 (4.8-9.8)** | **9.6 (6.3-16.1)** | **0.004** |
| C-reactive protein (mg/L) | **11 (5-35)** | **29 (9-61)** | **0.005** |
| Bilirubin (mg/dL) | **4.2 (1.9-8.3)** | **12.9 (4.6-27.3)** | **<0.001** |
| Prothrombin time (international normalized ratio) | **1.5 (1.4-1.9)** | **1.8 (1.5-2.3)** | **0.017** |
| Albumin (g/dL) | 3.0 ± 0.7 | 3.1 ± 0.7 | 0.835 |
| Creatinine (mg/dL) | **0.8 (0.6-1.1)** | **1.6 (0.9-2.6)** | **<0.001** |
| Sodium (mmol/L) | 136 (132-139) | 134 (128-140) | 0.318 |
| Platelets (10^9^/L) | 107 ± 69 | 130 ± 82 | 0.119 |
| *Scores* |  |  |  |
| MELD | **18 (14-21)** | **29 (23-34)** | **<0.001** |
| MELD Na | **20 (16-25)** | **31 (26-36)** | **<0.001** |
| Child-Pugh score | **9.4 ± 1.9** | **11.0 ± 1.7** | **<0.001** |
| CLIF-OFs | **7 (6-8)** | **10 (9-11)** | **<0.001** |
| *Outcome* |  |  |  |
| 28-day mortality (n, %) | 7 (11.9) | 12 (25.5) | 0.079 |
| 90-day mortality (n, %) | **13 (22.0)** | **23 (48.9)** | **0.004** |

Continues data are expressed as mean ± standard deviation or median (Q1-Q3), as appropriate. Categorical data are expressed as n(%). ACLF, acute on chronic liver failure; CLIF-OFs, chronic liver failure-organ failure score; HCV, hepatitis C virus; MELD, model for end-stage liver disease.

**Table S3. Patient characteristics stratified by survivors or non-survivors at 28-day and 90-day – validation cohort**

|  | 28-day | | | 90-day | | |
| --- | --- | --- | --- | --- | --- | --- |
|  | Survivors  (N=87) | Non-survivors  (N=19) | *p*  values | Survivors  (N=70) | Non-survivors  (N=36) | *p*  values |
| *Biomarker* |  |  |  |  |  |  |
| RIPK3(pg/ml) | **3989 (2073-10318)** | **8595**  **(4024-12658)** | **0.037** | **3261 (2003-9525)** | **7958 (4145-13453)** | **0.001** |
| Nucleosomes (ng/ml) | 0.001 (0.001-44) | 0.001 (0.001-0.001) | 0.183 | 0.001 (0.001-30) | 0.001 (0.001-71) | 0.112 |
| cK18 (U/L) | 704 (342-2289) | 847 (486-4572) | 0.215 | 651 (341-2230) | 894 (399-2953) | 0.328 |
| K18 (U/L) | 734 (292-5390) | 987 (219-9323) | 0.660 | 604 (258-5154) | 1407 (364-7832) | 0.208 |
| cK18:K18 ratio | 0.83 (0.50-1.40) | 0.73 (0.54-2.59) | 0.584 | 0.85 (0.53-2.19) | 0.69 (0.45-2.18) | 0.408 |
| Age (years) | 51 (43-60) | 49 (43-55) | 0.755 | 51 (45-61) | 50 (42-59) | 0.946 |
| Male (n, %) | 50 (57.5) | 12 (63.2) | 0.799 | 38 (54.3) | 24 (66.7) | 0.298 |
| *Etiology (n, %)* |  |  |  |  |  |  |
| Alcohol | 52 (59.8) | 13 (68.4) | 0.606 | 40 (57.1) | 25 (69.4) | 0.293 |
| Alcohol + HCV | 9 (10.3) | 2 (10.5) | >0.999 | 7 (10.0) | 4 (11.1) | >0.999 |
| HCV | 7 (8.0) | - | - | 5 (7.1) | 2 (5.6) | >0.999 |
| Non-alcoholic steatohepatitis | 6 (6.9) | 1 (5.3) | >0.999 | 6 (8.6) | 1 (2.8) | 0.419 |
| Other | 13 (14.9) | 3 (15.8) | >0.999 | 12 (17.1) | 4 (11.1) | 0.569 |
| Active alcoholism at enrollment  (n, %) | 64 (73.6) | 14 (73.7) | >0.999 | 52 (74.3) | 26 (72.2) | 0.820 |
| Ascites (n, %) | 49 (56.3) | 10 (52.6) | 0.803 | 38 (54.3) | 21 (58.3) | 0.837 |
| Gastrointestinal bleeding (n, %) | 24 (27.6) | 3 (15.8) | 0.389 | 18 (25.7) | 9 (25.0) | >0.999 |
| Bacterial infection (n, %) | 25 (28.7) | 9 (47.4) | 0.173 | **17 (24.3)** | **16 (44.4)** | **0.046** |
| *Organ failure (n, %)* |  |  |  |  |  |  |
| Liver | 25 (28.7) | 7 (36.8) | 0.583 | 18 (25.7) | 14 (38.9) | 0.185 |
| Kidney | 17 (19.5) | 8 (42.1) | 0.069 | 14 (20.0) | 11 (30.6) | 0.237 |
| Brain | 11 (12.6) | - | - | **4 (5.7)** | **7(19.4)** | **0.042** |
| Coagulation | 8 (9.2) | 3 (15.8) | 0.411 | 6 (8.6) | 5 (13.9) | 0.504 |
| Cardiac^1^ | **7 (8.0)** | **4 (21.1)** | **0.033** | 4 (5.7) | 7 (19.4) | 0.268 |
| Respiratory | 4 (4.6) | 1 (5.3) | >0.999 | 2 (2.9) | 3 (8.3) | 0.334 |
| *Laboratory values* |  |  |  |  |  |  |
| White blood cell (x10^9^/L) | 8.0 (4.9-11.0) | 8.8 (5.1-15.5) | 0.250 | 7.8 (4.8-10.7) | 8.6 (6.1-15.1) | 0.118 |
| C-reactive protein (mg/L) | 19 (5-47) | 19 (7-57) | 0.796 | 16 (5-40) | 24 (7-59) | 0.255 |
| Bilirubin (mg/dL) | 5.8 (2.1-16.3) | 4.4 (0.9-8.5) | 0.187 | **4.6 (1.9-12.5)** | **9.9 (5.8-24.9)** | **0.009** |
| Prothrombin time  (international normalized ratio) | 1.7 (1.4-2.0) | 1.5 (1.3-1.7) | 0.399 | **1.6 (1.4-1.9)** | **1.8 (1.5-2.3)** | **0.027** |
| Albumin (g/dL) | 3.1 ± 0.7 | 2.9 ± 0.7 | 0.260 | 3.1 ± 0.7 | 3.0 ± 0.8 | 0.462 |
| Creatinine (mg/dL) | 0.9 (0.7-1.4) | 0.8 (0.4-1.1) | 0.145 | 0.9 (0.7-1.5) | 1.1 (0.8-1.6) | 0.097 |
| Sodium (mmol/L) | 135 (132-139) | 132 (129-138) | 0.066 | 135 (132-139) | 133 (129-138) | 0.173 |
| Platelets (10^9^/L) | 116 ± 73 | 122 ± 89 | 0.774 | 118 ± 75 | 115 ± 78 | 0.827 |
| *Scores* |  |  |  |  |  |  |
| MELD | **21 (16-28)** | **24 (20-33)** | **0.040** | **19 (16-27)** | **26 (21-30)** | **0.013** |
| MELD Na | **24 (18-30)** | **28 (23-36)** | **0.038** | **23 (17-30)** | **28 (23-32)** | **0.002** |
| Child-Pugh score | 10.1 ± 1.9 | 10.4 ± 2.3 | 0.520 | **9.7 ± 1.9** | **10.8 ± 2.0** | **0.008** |
| CLIF-OFs | 8 (7-10) | 9 (7-11) | 0.079 | **8 (6-9)** | **10 (8-11)** | **<0.001** |

Data are expressed as mean ± SD or median (Q1-Q3).

cK18, caspase-cleaved keratin 18; CLIF-OFs, chronic liver failure-organ failure score; HCV, hepatitis C virus; K18, keratin 18; MELD, model for end-stage liver disease; RIPK3, receptor interacting protein kinase 3

**Table S4. Clinical characteristics of patients whose liver biopsies were evaluated by immunohistochemistry**

| Patient Number | ACLF | Age | Sex | Etiology | Precipitating event |
| --- | --- | --- | --- | --- | --- |
| 1. | No | 40 | Male | Alcohol | Active alcoholism |
| 2. | No | 52 | Female | Alcohol | Active alcoholism |
| 3. | No | 50 | Male | Alcohol | Active alcoholism |
| 4. | No | 44 | Female | Alcohol | Active alcoholism |
| 5. | Yes | 34 | Male | Alcohol | Active alcoholism |
| 6. | Yes | 56 | Male | Alcohol | Active alcoholism |
| 7. | Yes | 54 | Female | Alcohol | Active alcoholism |
| 8. | Yes | 30 | Female | Alcohol | Active alcoholism |

ACLF, acute on chronic liver failure.

**Table S5. List of antibodies for immunohistochemistry**

| **Antibody** | **Company** | **Clone** | **Catalogue Number** |
| --- | --- | --- | --- |
| Anti-RIPK3 (Rabbit Polyclonal) | Gentex | polyclonal | GTX31389 |
| Anti-RIPK3 (Rabbit Polyclonal) | Novus biologicals | polyclonal | NBP1-77299 |
| Anti-RIPK1 (Rabbit Polyclonal) | Gentex | polyclonal | GTX31684 |
| Anti-cleaved Caspase 8 (Rabbit polyclonal) | Novus Biologicals | polyclonal | NB100-56116 |
| Anti-cleaved Caspase 3 (Asp175) (rabbit monoclonal) | RD Systems | 269518 | MAB835 |
| Anti-pMLKL (pS358) (rabbit monoclonal) | Abcam | EPR9514 | ab187091 |
| Anti-pMLKL [pS345] | Novus Biologicals | JM92-37 | NBP2-66953 |
| Rabbit IgG isotype Ctrl | Novus Biologicals | Polyclonal | NB810-56910 |

**Table S6. Performance of biomarkers and clinical scores in predicting progression to ACLF, 28 and 90-day mortality**

|  | Progression to ACLF  AUROC (95% confidence interval)  (derivation cohort n=170)  (validation cohort n=59) | 28-day mortality  AUROC (95% confidence interval)  (derivation cohort n=251)  (validation cohort n=106) | 90-day mortality  AUROC (95% confidence interval)  (derivation cohort n=251)  (validation cohort n=106) |
| --- | --- | --- | --- |
| RIPK3 (derivation cohort) | 0.650 (0.562-0.738) | 0.768 (0.679-0.858) | 0.676 (0.595-0.758) |
| RIPK3 (validation cohort) | 0.744 (0.593-0.895) | 0.653 (0.530-0.776) | 0.696 (0.593-0.799) |
| Nucleosomes (validation cohort) | 0.688 (0.554-0.823) | 0.582 (0.478-0.686) | 0.579 (0.477-0.682) |
| cK18 (validation cohort) | 0.730 (0.568-0.892) | 0.592 (0.456-0.728) | 0.559 (0.444-0.673) |
| K18 (validation cohort) | 0.702 (0.527-0.877) | 0.533 (0.374-0.692) | 0.575 (0.458-0.692) |
| cK18:K18 ratio (validation cohort) | 0.527 (0.368-0.686) | 0.541 (0.387-0.694) | 0.550 (0.430-0.669) |
| Child-Pugh score (derivation cohort) | 0.735 (0.637-0.832) | 0.762 (0.663-0.860) | 0.750 (0.672-0.827) |
| Child-Pugh score (validation cohort) | 0.688 (0.537-0.840) | 0.549 (0.397-0.702) | 0.643 (0.532-0.753) |
| MELD (derivation cohort) | 0.700 (0.608-0.792) | 0.781 (0.687-0.874) | 0.781 (0.709-0.853) |
| MELD (validation cohort) | 0.744 (0.595-0.894) | 0.650 (0.516-0.783) | 0.688 (0.582-0.794) |
| MELD Na (derivation cohort) | 0.725 (0.637-0.813) | 0.777 (0.685-0.869) | 0.785 (0.714-0.855) |
| MELD Na (validation cohort) | 0.693 (0.541-0.894) | 0.654 (0.521-0.786) | 0.686 (0.573-0.800) |
| CLIF-OFs (derivation cohort) | 0.697 (0.602-0.793) | 0.757 (0.649-0.865) | 0.711 (0.628-0.794) |
| CLIF-OFs (validation cohort) | 0.724 (0.585-0.864) | 0.628 (0.499-0.756) | 0.730 (0.634-0.826) |
| CLIF-C AD score (derivation cohort) | 0.747 (0.668-0.826) | 0.748 (0.603-0.893) | 0.774 (0.673-0.875) |
| CLIF-C AD score (validation cohort) | 0.597 (0.434-0.761) | 0.688 (0.547-0.829) | 0.686 (0.573-0.800) |
| CLIF-C ACLF score  (derivation cohort) | - | 0.857 (0.775-0.939) | 0.744 (0.634-0.854) |
| CLIF-C ACLF score (validation cohort) | - | 0.634 (0.483-0.785) | 0.720 (0.616-0.824) |

AD, acute decompensation; ACLF, acute on chronic liver failure; AUROC, area under receiver operating characteristic; cK18, caspase-cleaved keratin 18; CLIF-C, chronic liver failure-consortium; CLIF-OFs, chronic liver failure-organ failure score; K18, keratin 18; MELD, model for end-stage liver disease; RIPK3, receptor interacting protein kinase 3

**Table S7. Multivariate analysis of factors associated with 28-day and 80-day mortality**

| 28-day mortality (derivation cohort) | | | | |
| --- | --- | --- | --- | --- |
| Parameter | Hazard ratio (95% confidence interval) | *p* value | Hazard ratio (95% confidence interval) | *p* value |
| Age | 1.005 (0.975-1.035) | 0.765 | - |  |
| Gender (male) | 0.772 (0.370-1.611) | 0.490 | - |  |
| RIPK3 / 1000 | **1.082 (1.057-1.109)** | **0.001** | **1.035 (1.002-1.068)** | **0.037** |
| WBC | **1.129 (1.084-1.177)** | **0.001** | **1.081 (1.026-1.138)** | **0.003** |
| ACLF grade | **2.813 (1.843-4.293)** | **0.001** | 1.243 (0.654-2.360) | 0.507 |
| MELD | **1.139 (1.090-1.191)** | **0.001** | 1.059 (0.992-1.131) | 0.084 |

| 90-day mortality (derivation cohort) | | | | |
| --- | --- | --- | --- | --- |
| Parameter | Hazard ratio (95% CI) | *p* value | Hazard ratio (95% CI) | *p* value |
| Age | 0.996 (0.975-1.017) | 0.696 | - |  |
| Gender (male) | 0.629 (0.371-1.068) | 0.086 | - |  |
| RIPK3 / 1000 | **1.065 (1.044-1.087)** | **0.001** | 1.012 (0.986-1.039) | 0.364 |
| WBC | **1.127 (1.091-1.164)** | **0.001** | **1.085 (1.045-1.126)** | **0.001** |
| ACLF grade | **2.248 (1.654-3.055)** | **0.001** | 0.925 (0.580-1.475) | 0.743 |
| MELD | **1.143 (1.105-1.183)** | **0.001** | **1.113 (1.058-1.171)** | **0.001** |

**Table S8. Biomarkers (baseline) stratified by patient group (validation cohort)**

|  | RIPK3 (pg/ml)  Median (IQR) | Nucleosomes (ng/ml)  Median (IQR) | cK18 (U/L)  Median (IQR) | K18 (U/L)  Median (IQR) | cK18:K18 ratio  Median (IQR) |
| --- | --- | --- | --- | --- | --- |
| Healthy controls  (n=21) | **322 (136-493)** | - | - | - | - |
| Stable cirrhosis  (n-42) | **445 (320-565)** | - | - | - | - |
| All decompensated  (n=106) | **4559 (2302-10604)** | 0.001 (0.001-41) | 715 (365-2514) | 820 (288-5881) | 0.80 (0.50-2.19) |
| *p* value | **0.001** | - | - | - | - |
| No ACLF  (n=59) | 2804 (1665-5551) | 0.001 (0.001-37) | 486 (329-1745) | 441 (235-1890) | 0.89 (0.58-2.50) |
| ACLF grade 1  (n=19) | 5055 (2807-11753) | 0.001 (0.001-0.001) | 700 (346-1366) | 810 (347-2526) | 0.71 (0.50-2.44) |
| ACLF grade 2 or 3  (n=28) | 11438 (7639-23940) | 25.5 (0.001-92) | 2293 (709-6205) | 5957 (1380-11580) | 0.53 (0.34-1.91) |
| *p* value | **0.001** | **0.022** | **0.001** | **0.001** | 0.081 |
| AD throughout  (n=41) | **2513 (1563-3683)** | **0.001 (0.001-0.001)** | **441 (311-845)** | **355 (218-923)** | 0.89 (0.57-2.90) |
| AD to ACLF  (n=18) | **5597 (1894-9972)** | **32 (0.001-70)** | **1905 (363-6840)** | **1839 (281-10531)** | 0.90 (0.60-1.9) |
| *p* value | **0.003** | **0.003** | **0.005** | **0.014** | 0.750 |

ACLF, acute on chronic liver failure; AD, acute decompensation; cK18, caspase-cleaved keratin 18; K18, keratin 18; IQR, interquartile range; RIPK3, receptor interacting protein kinase 3

**Table S9. Biomarkers (baseline) stratified by the Predisposition, Injury and Response (validation cohort)**

|  | RIPK3 (pg/ml)  Median (IQR) | Nucleosomes (μg/ml)  Median (IQR) | cK18 (U/L)  Median (IQR) | K18 (U/L)  Median (IQR) | cK18:K18 ratio  Median (IQR) |
| --- | --- | --- | --- | --- | --- |
| ***Predisposition*** | | | | | |
| Age <50 | 5661 (2898-12439) | 0.001 (0.001-54) | 772 (350-2937) | 966 (324-8485) | 0.68 (0.40-2.50) |
| Age ≥50 | 3659 (1985-9651) | 0.001 (0.001-38) | 702 (368-2331) | 704 (272-3163) | 0.84 (0.54-1.48) |
| *p* value | 0.063 | 0.486 | 0.674 | 0.399 | 0.367 |
| Male | 3830 (2287-7078) | 0.001 (0.001-0.001) | 715 (388-2898) | 846 (254-7092) | 0.70 (0.46-2.48) |
| Female | 5706 (2302-12052) | 0.001 (0.001-49) | 742 (341-2331) | 752 (296-5334) | 0.84 (0.52-1.82) |
| *p* value | 0.162 | 0.076 | 0.613 | 0.945 | 0.846 |
| Etiology - No Alcohol | **3270 (2059-8627)** | 0.001 (0.001-38) | 583 (352-1877) | **570 (199-2034)** | 0.87 (0.54-3.16) |
| Etiology - Alcohol | **5642 (2586-11897)** | 0.001 (0.001-43) | 847 (368-2859) | **996 (346-8821)** | 0.69 (0.48-1.54) |
| *p* values | **0.047** | 0.870 | 0.180 | **0.031** | 0.187 |
| Etiology – No HCV | 4682 (2368-10635) | 0.001 (0.001-41) | 726 (369-2455) | 882 (277-6111) | 0.85 (0.52-2.43) |
| Etiology – HCV | 3671 (2073-9815) | 0.001 (0.001-107) | 370 (261-2689) | 667 (458-2164) | 0.50 (0.31-0.78) |
| *p* values | 0.746 | 0.514 | 0.334 | 0.920 | 0.126 |
| Etiology – No HCV+Alcohol | 4383 (2302-10880) | 0.001 (0.001-40) | 704 (364-2455) | 734 (292-5390) | 0.78 (0.50-2.43) |
| Etiology –HCV+Alcohol | 4946 (1845-9832) | 0.001 (0.001-152) | 760 (468-4572) | 1247 (268-8511) | 0.95 (0.54-1.40) |
| *p* values | 0.499 | 0.466 | 0.637 | 0.728 | 0.878 |
| Etiology – No NASH | 4946 (2300-10726) | 0.001 (0.001-43) | 760 (364-2752) | **965 (326-7230)** | 0.75 (0.50-2.08) |
| Etiology – NASH | 2802 (2368-3247) | 0.001 (0.001-0.001) | 473 (381-1172) | **235 (98-734)** | 1.38 (0.60-5.85) |
| *p* values | 0.068 | 0.093 | 0.293 | **0.020** | 0.126 |
| Etiology – No Others | 4559 (2352-10626) | 0.001 (0.001-42) | 772 (369-2514) | 966 (316-6663) | 0.72 (0.48-1.48) |
| Etiology – Others | 4794 (1562-10333) | 0.001 (0.001-35) | 576 (347-2400) | 446 (86-4083) | 1.02 (0.58-3.25) |
| *p* values | 0.543 | 0.969 | 0.419 | 0.086 | 0.097 |
| No Ascites | 4024 (1908-9832) | 0.001 (0.001-22) | 583 (381-1745) | 734 (258-2540) | 0.95 (0.50-1.72) |
| Ascites | 4946 (2565-10726) | 0.001 (0.001-65) | 904 (346-4118) | 882 (322-8406) | 0.68 (0.50-2.44) |
| *p* values | 0.375 | 0.093 | 0.246 | 0.287 | 0.980 |
| ***Injury*** | | | | | |
| No Infection (n=72) | 4204 (2130-10416) | 0.001 (0.001-41) | 651 (366-2380) | 704 (293-6873) | 0.85 (0.53-1.99) |
| Infection (n=34) | 5126 (2829-11749) | 0.001 (0.001-58) | 894 (358-2705) | 935 (272-5570) | 0.69 (0.42-2.43) |
| *p* values | 0.390 | 0.355 | 0.878 | 0.894 | 0.640 |
| No active alcoholism (n=28) | 3258 (2083-8740) | 0.001 (0.001-38) | 537 (365-1569) | **514 (126-1373)** | **1.03 (0.61-4.26)** |
| Active alcoholism (n=78) | 5146 (2352-10993) | 0.001 (0.001-44) | 821 (360-2980) | **983 (341-8432)** | **0.69 (0.48-1.30)** |
| *p* values | 0.190 | 0.873 | 0.155 | **0.012** | **0.019** |
| No GI-bleeding (n=79) | 4276 (2195-10593) | 0.001 (0.001-44) | 704 (346-2752) | 667 (292-7230) | 0.87 (0.52-2.44) |
| GI bleeding (n=27) | 6318 (2658-10635) | 0.001 (0.001-37) | 785 (443-1887) | 1352 (258-3015) | 0.68 (0.42-1.36) |
| *p* values | 0.423 | 0.923 | 0.996 | 0.734 | 0.363 |
| ***Response*** | | | | | |
| White blood cell  <8 (x10^9^/L) | **3302 (2011-6264)** | **0.001 (0.001-0.001)** | **556 (346-1806)** | **458 (249-1463)** | **0.87 (0.52-2.59)** |
| White blood cell  8-12 (x10^9^/L) | **4968 (2243-9972)** | **0.001 (0.001-48)** | **651 (357-2409)** | **830 (265-3530)** | **0.94 (0.63-2.19)** |
| White blood cell  >12 (x10^9^/L) | **10843(6695-23585)** | **28 (0.001-76)** | **2293 (606-11974)** | **7564 (1137-15651)** | **0.52 (0.33-2.05)** |
| *p* values | **<0.001** | **0.039** | **0.008** | **0.001** | **0.040** |
| C-reactive protein  <10 (mg/L) | **3253 (1908-6318)** | 0.001 (0.001-43) | 760 (369-1745) | **458 (219-2164)** | **0.99 (0.60-2.81)** |
| C-reactive protein  10-20 (mg/L) | **4488 (2407-10921)** | 0.001 (0.001-59) | 468 (391-705) | **441 (175-1034)** | **1.02 (0.59-3.33)** |
| C-reactive protein  >20 (mg/L) | **7332 (2731-13919)** | 0.001 (0.001-41) | 1623 (352-3796) | **2051 (394-10003)** | **0.55 (0.41-1.15)** |
| *p* values | **0.029** | 0.983 | 0.230 | **0.008** | **0.008** |

cK18, caspase-cleaved keratin 18; GI, gastrointestinal; HCV, hepatitis C virus; IQR, interquartile range; K18, keratin 18; NASH, non-alcoholic steatohepatitis; RIPK3, receptor interacting protein kinase 3.

**Table S10. Plasma biochemistry levels of sham, BDL, BDL + LPS and BDL + NEC-1 + LPS**

|  | Sham | BDL | BDL + LPS | BDL + NEC-1 + LPS |
| --- | --- | --- | --- | --- |
| ALT (IU/L) | 35.4 ± 3.7 | 68.4 ± 11.6^$^ | 79.4 ± 12.8^$^ | 74.1 ± 7.4^$$^ |
| AST (IU/L) | 65.0 ± 2.0 | 252.5 ± 31.3^$^ | 412.9 ± 39.4^$$,^ * | 290.5 ± 35.5^$$, #^ |
| Albumin (g/L) | 28.4 ± 2.6 | 21.6 ± 1.8 | 19.1 ± 1.3 | 23.0 ± 0.8^#^ |
| Bilirubin (μmol/L) | 0.9 ± 0.4 | 201.6 ± 38.9^$^ | 167.6 ± 15.1^$$^ | 215.8 ± 10.1^$$, #^ |
| Creatinine (μmol/L) | 21.6 ± 3.6 | 26.1 ± 2.0 | 48.0 ± 8.1 | 25.2 ± 2.0^#^ |
| Urea (mmol/L) | 6.1 ± 0.7 | 5.6 ± 0.6 | 9.7 ± 1.2 | 5.8 ± 0.4^##^ |
| Glucose (mmol/L) | 12.1 ± 1.9 | 6.4 ± 1.3^$^ | 4.6 ± 1.1^$$^ | 4.7 ± 0.9^$$^ |
| Ammonia (μg/dl) | 58.8 ± 5.1 | 169.2 ± 39.3 | 293.5 ± 59.7^$$^ | 233.4 ± 70.6 |

Data are expressed as mean ± standard error of mean.

ALT, alanine aminotransferase; AST, aspartate transaminase; BDL, bile duct ligation; LPS, lipopolysaccharide; NEC-1, necrostatin-1.

^$^*p*<0.05 and ^$$^*p*<0.01 compared to the sham group.

**P*<0.05 compared to the BDL group.

^#^*P*<0.05 and ^##^*P*<0.01 compared to the BDL + LPS group.
