# Supplementary figures and images for: The role of RIPK1 mediated cell death in acute on chronic liver failure
Source: Cell Death Dis. 2021 Dec 17;13(1):5. doi: 10.1038/s41419-021-04442-9 (PMC8683430; doi:10.1038/s41419-021-04442-9)

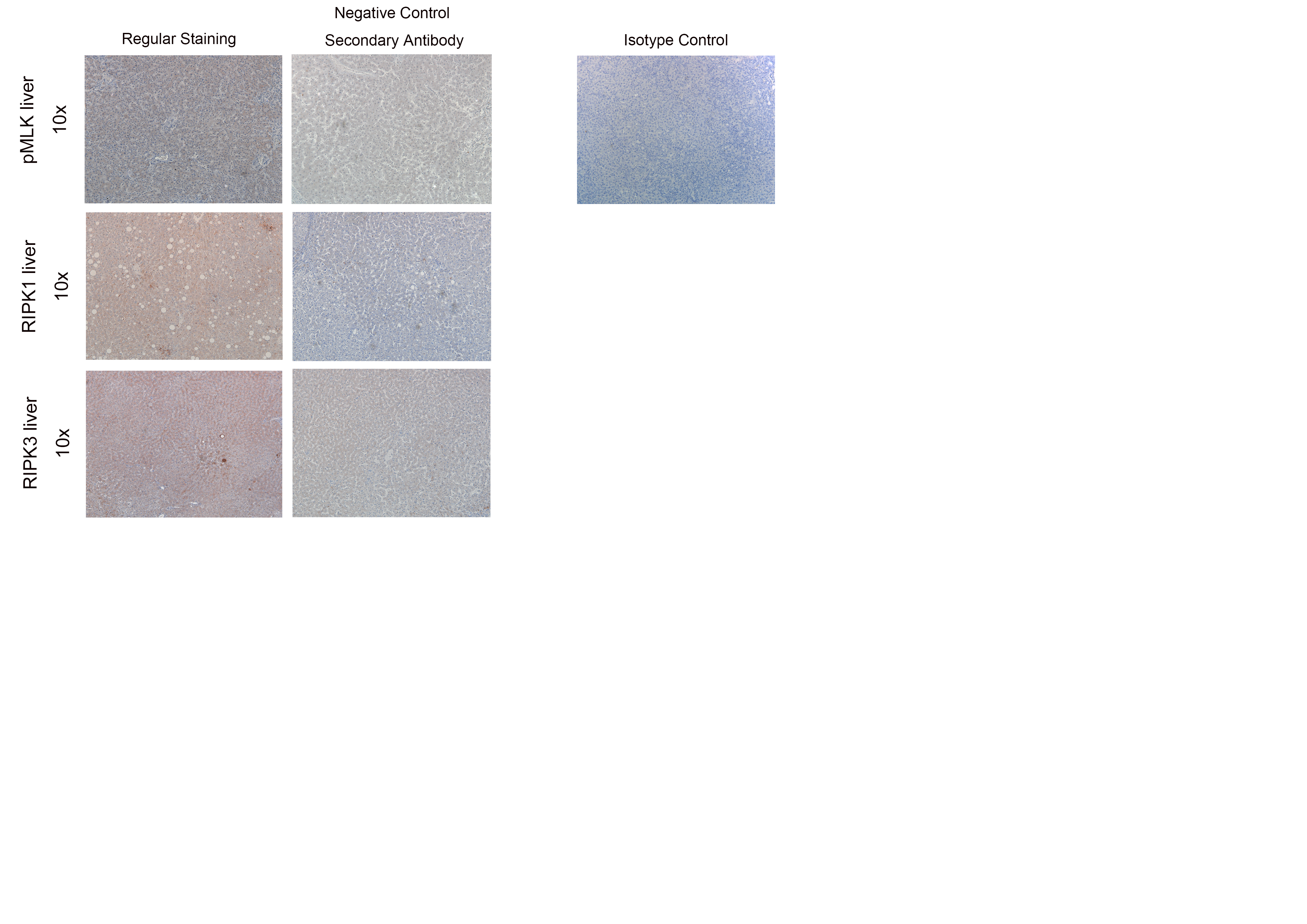

Supplement: Supplementary file 4 — Figure S2 [file 41419_2021_4442_MOESM4_ESM.tif]

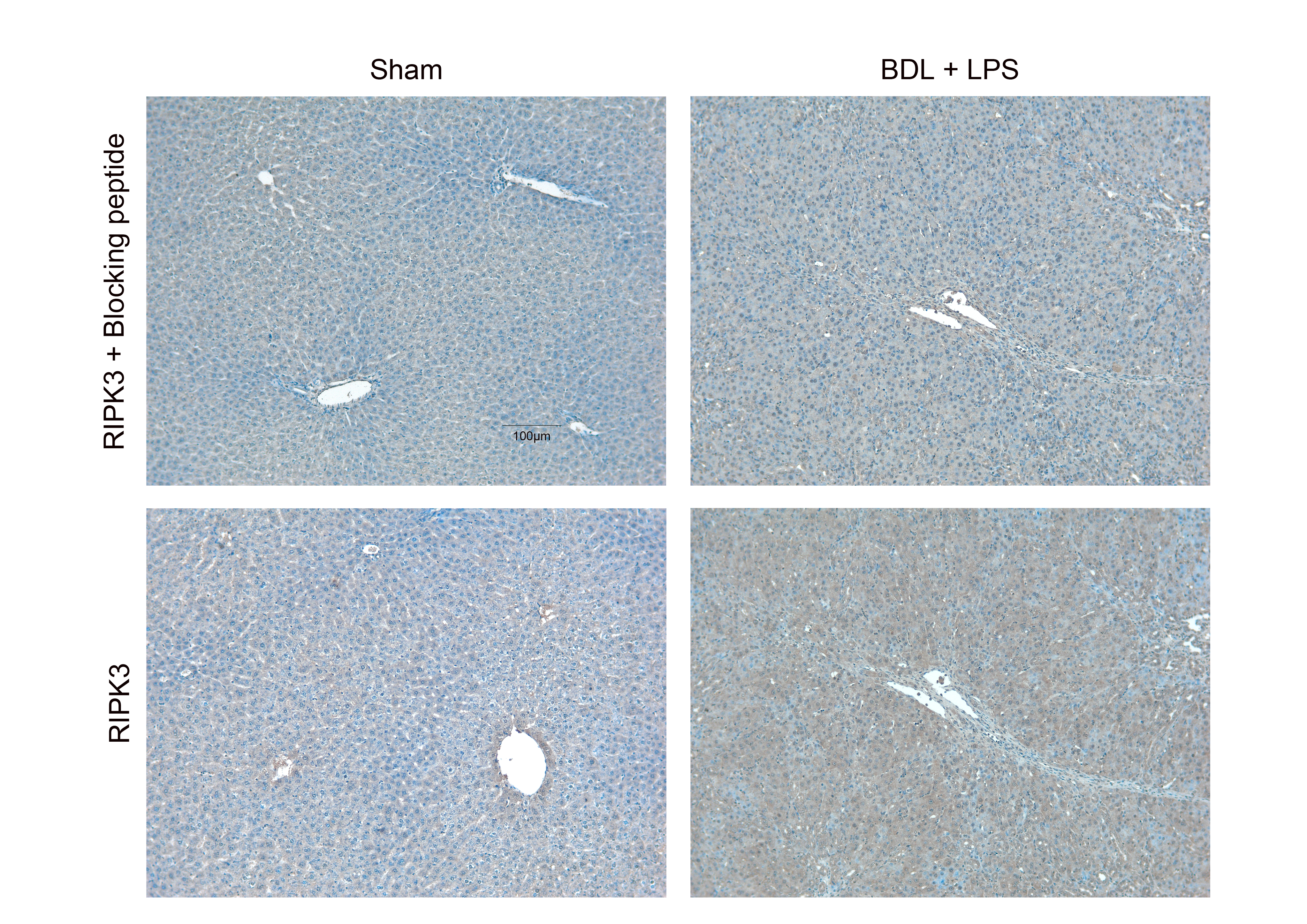

Supplement: Supplementary file 5 — Figure S3 [file 41419_2021_4442_MOESM5_ESM.tif]

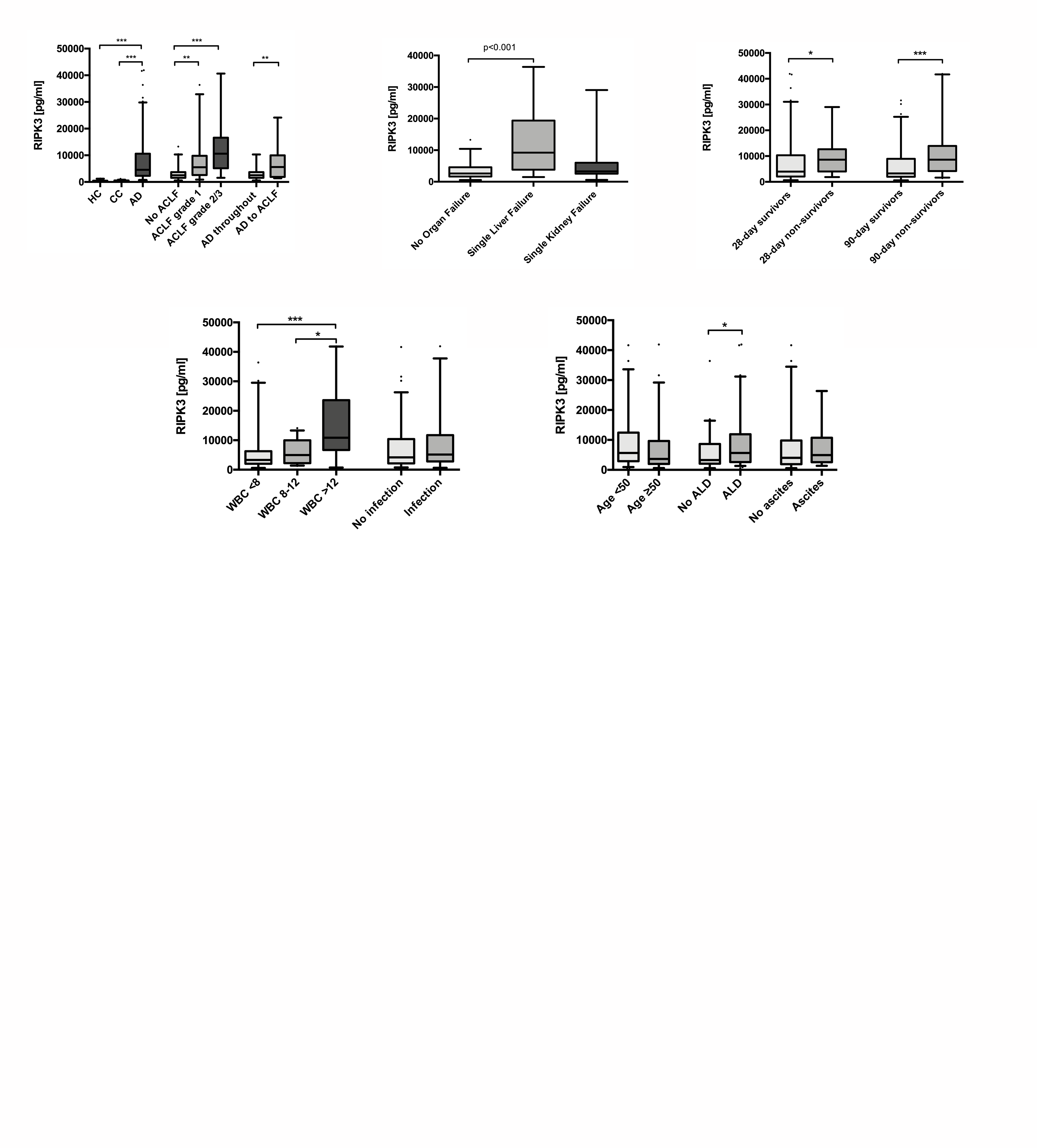

Supplement: Supplementary file 6 — Figure S4 [file 41419_2021_4442_MOESM6_ESM.tif]

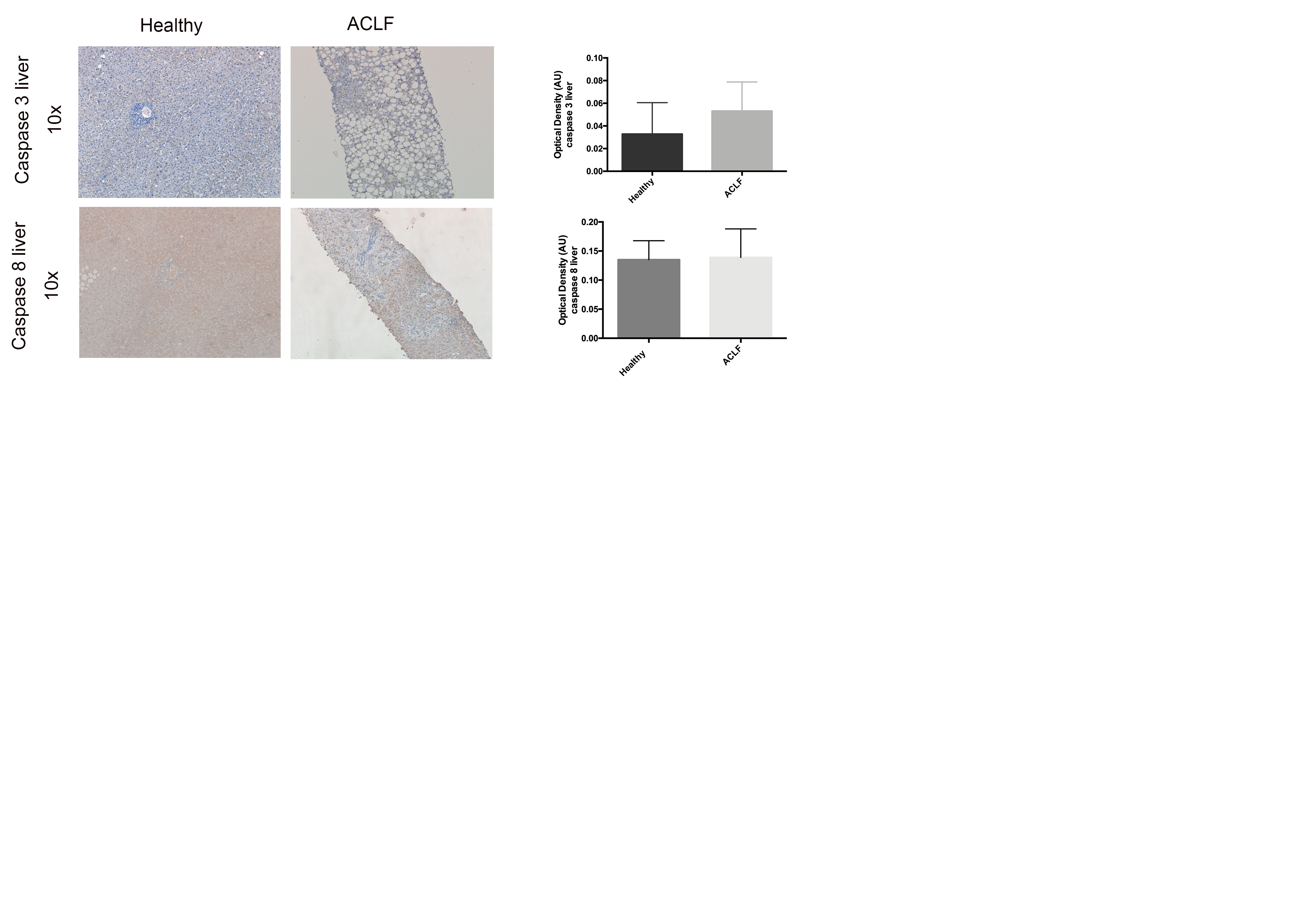

Supplement: Supplementary file 7 — Figure S5 [file 41419_2021_4442_MOESM7_ESM.tif]

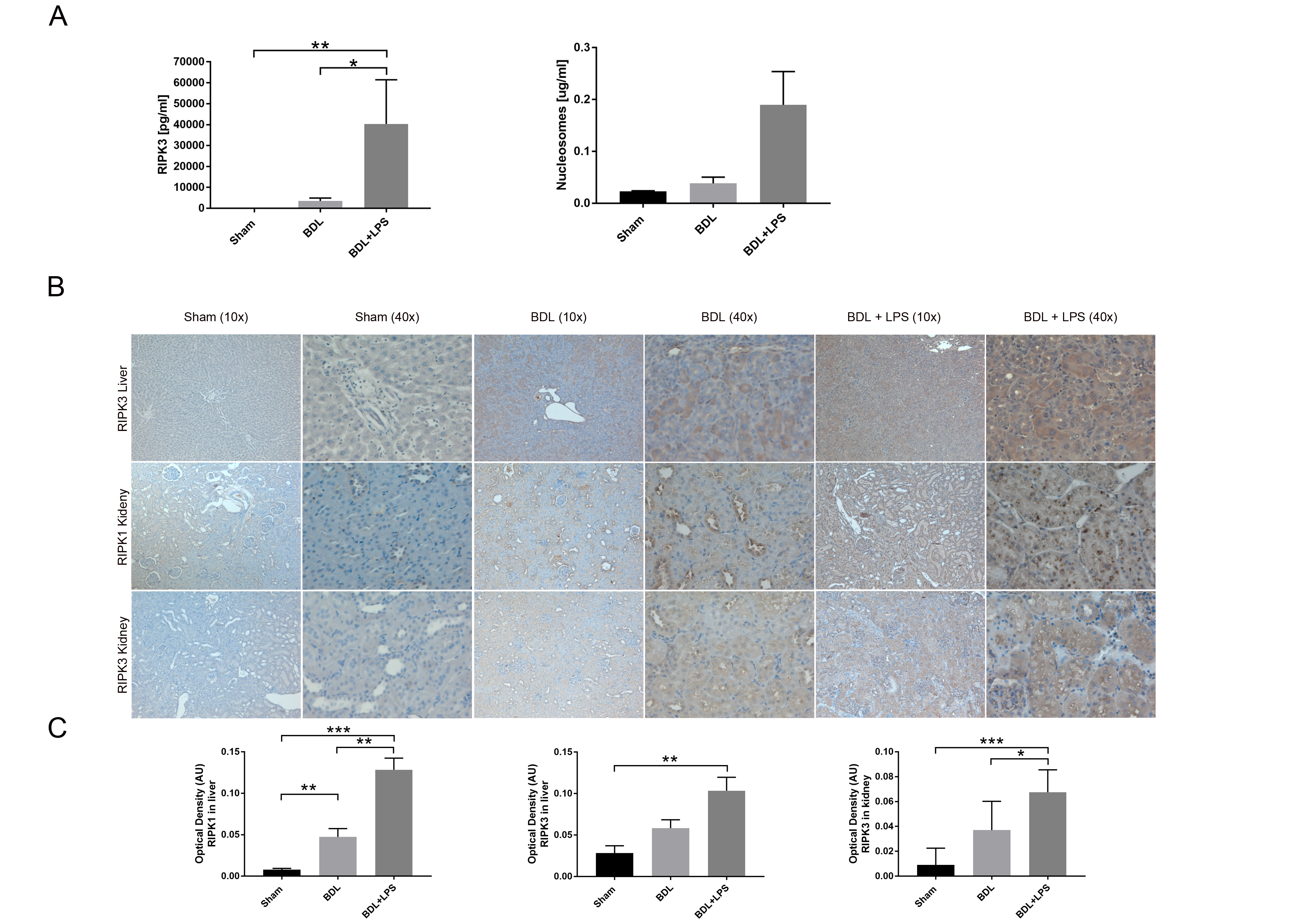

Supplement: Supplementary file 8 — Figure S6 [file 41419_2021_4442_MOESM8_ESM.tif]

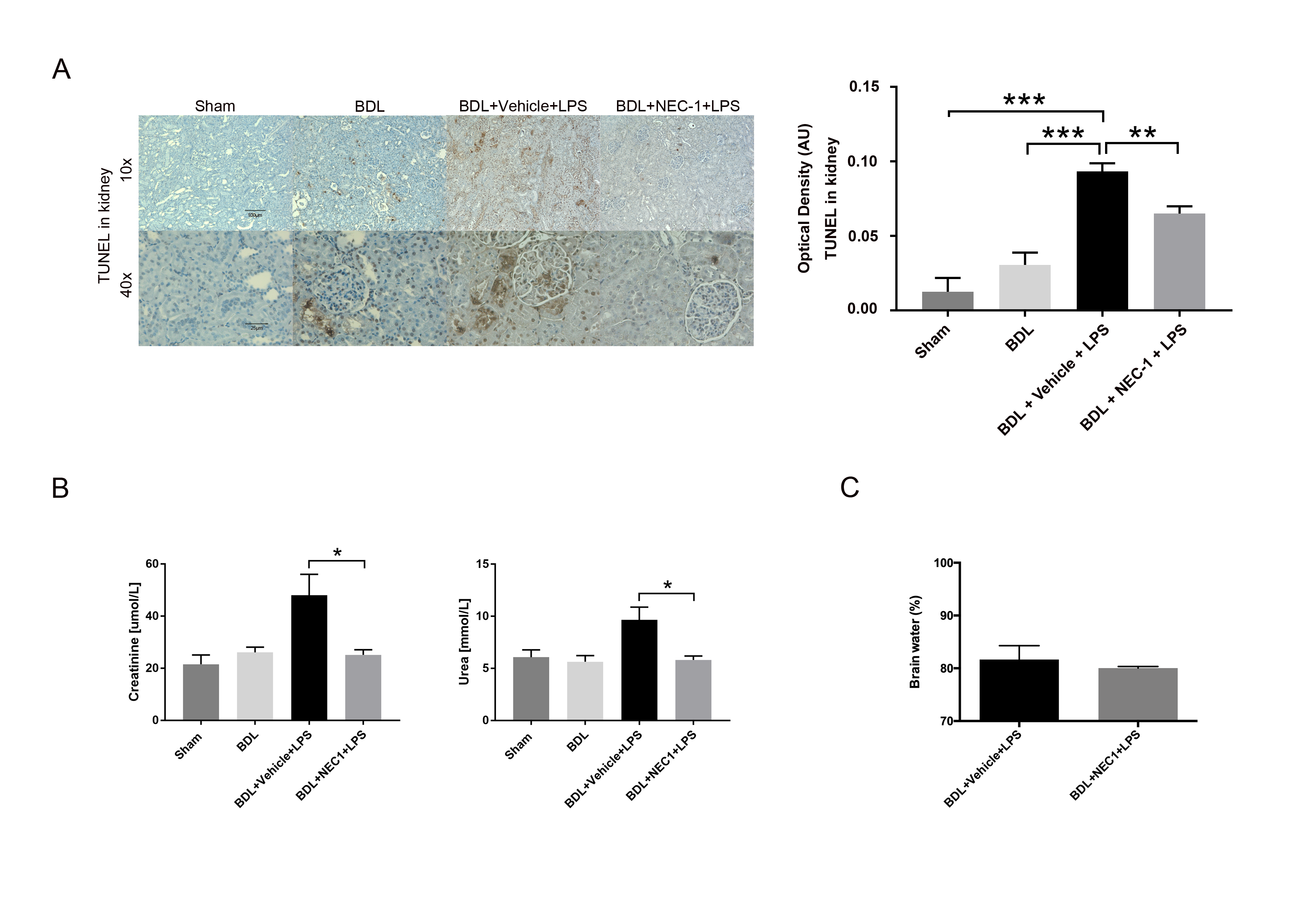

Supplement: Supplementary file 9 — Figure S7 [file 41419_2021_4442_MOESM9_ESM.tif]
